# Astaxanthin mitigates doxorubicin-induced cardiotoxicity via inhibiting ferroptosis and autophagy: a study based on bioinformatic analysis and *in vivo*/*vitro* experiments

**DOI:** 10.3389/fphar.2025.1524448

**Published:** 2025-01-21

**Authors:** Bowen Yin, Jingyi Ren, Xuanyi Liu, Yadong Zhang, Jinshi Zuo, Rui Wen, Huanting Pei, Miaomiao Lu, Siqi Zhu, Zhenao Zhang, Ziyi Wang, Yanyi Zhai, Yuxia Ma

**Affiliations:** ^1^ Department of Nutrition and Food Hygiene, School of Public Health, Hebei Medical University, Hebei Key Laboratory of Environment and Human Health, Shijiazhuang, China; ^2^ Undergraduate of College of Public Health, Hebei Medical University, Shijiazhuang, China

**Keywords:** astaxanthin, doxorubicin, cardiotoxicity, ferroptosis, autophagy, rat

## Abstract

**Background:**

Doxorubicin (DOX), a widely employed chemotherapeutic agent in cancer treatment, has seen restricted use in recent years owing to its associated cardiotoxicity. Current reports indicate that doxorubicin-induced cardiotoxicity (DIC) is a complex phenomenon involving various modes of cell death. Astaxanthin (ASX), a natural carotenoid pigment, has garnered significant attention for its numerous health benefits. Recent studies have shown that ASX has a broad and effective cardiovascular protective effect. Our study aims to investigate the protective effects of ASX against DIC and elucidate its underlying mechanisms. This has substantial practical significance for the clinical application of DOX.

**Methods:**

Bioinformatic analyses were conducted using transcriptomic data from the gene expression omnibus (GEO) database to identify key mechanisms underlying DIC. Network pharmacology was employed to predict the potential pathways and targets through which ASX exerts its effects on DIC. *In vitro* experiments, following pretreatment with ASX, H9C2 cells were exposed to DOX. Cell viability, injury and the protein expression levels associated with ferroptosis and autophagy were assessed. In the animal experiments, rats underwent 4 weeks of gavage treatment with various doses of ASX, followed by intraperitoneal injections of DOX every 2 days during the final week. Histological, serum, and protein analyses were conducted to evaluate the effects of ASX on DIC.

**Results:**

The bioinformatics analysis revealed that ferroptosis and autophagy are closely associated with the development of DIC. ASX may exert an anti-DIC effect by modulating ferroptosis and autophagy. The experimental results show that ASX significantly mitigates DOX-induced myocardial tissue damage, inflammatory response, oxidative stress, and damage to H9C2 cells. Mechanistically, ASX markedly ameliorates levels of ferroptosis and autophagy both *in vitro* and *in vivo*. Specifically, ASX upregulates solute carrier family 7 member 11 (SLC7A11) and glutathione peroxidase 4 (GPX4), while downregulating the expression of transferrin receptor 1 (TFRC), ferritin heavy chain (FTH1) and ferritin light chain (FTL). Additionally, ASX enhances the expression of P62 and decreases levels of Beclin1 and microtubule-associated proteins light chain 3 (LC3).

**Conclusion:**

Our results indicate that ferroptosis and autophagy are critical factors influencing the occurrence and progression of DOX-induced cardiotoxicity. ASX can alleviate DIC by inhibiting ferroptosis and autophagy.

## 1 Introduction

As a kind of potent antitumor anthracycline antibiotic, doxorubicin (DOX) was widely used as a chemotherapy regimen for various malignant tumors such as leukemia, malignant lymphoma and so on. However, the existence of cardiotoxicity has largely hindered the application of DOX in actual clinical treatment. Doxorubicin-induced cardiotoxicity (DIC) typically manifests as eventual heart failure and left ventricular dysfunction (Chen et al., 2022). It is reported that more than a quarter of cancer patients develop congestive heart failure after receiving DOX treatment (Swain et al., 2003). The incidence of cardiovascular disease doubled in older lymphoma survivors treated with DOX compared to those treated without DOX (Nabhan et al., 2015). Currently, there are very few clinically available drugs to counteract DIC. Dexrazoxane is the only drug officially approved against DIC. Unfortunately, it has been reported that dexrazoxane itself also has strong side effects, which may cause secondary malignancy and even aggravate the myelosuppressive effects of DOX (Vejpongsa and Yeh, 2014). Therefore, identifying a safe and effective treatment strategy to inhibit DIC is of critical importance.

Previous studies have demonstrated that oxidative stress, inflammation, lipid peroxidation, and multiple forms of cell death are closely linked to the development of DIC (Abdel-Daim et al., 2017; Guo et al., 2020; Octavia et al., 2012). Multiple factors operate concurrently, overlap, and interact, significantly increasing the complexity of DIC (Christidi and Brunham, 2021). Several recent studies have suggested that ferroptosis plays a critical role in the pathogenesis of DIC (Cui et al., 2024; He et al., 2021; Li et al., 2022; Qiu et al., 2024). As a kind of novel programmed cell death, ferroptosis is mainly manifested by increased iron dependence of lipid peroxidation, excessive accumulation of ROS and damage of biofilm structure (Dixon et al., 2012). Meanwhile, current evidence indicates that DOX-induced cardiotoxicity is closely associated with autophagy, and that DIC can lead to dysregulation of autophagy levels in the heart (Bartlett et al., 2017). However, whether DOX induces or inhibits autophagy in cardiac tissues remains a contentious issue (Li et al., 2016; Park et al., 2016). Therefore, the overlap and crosstalk between ferroptosis and autophagy may represent a key mechanism underlying the development of DIC. Targeted modulation of ferroptosis and autophagy may offer a viable strategy for mitigating doxorubicin-induced cardiac injury.

**FIGURE 1:**
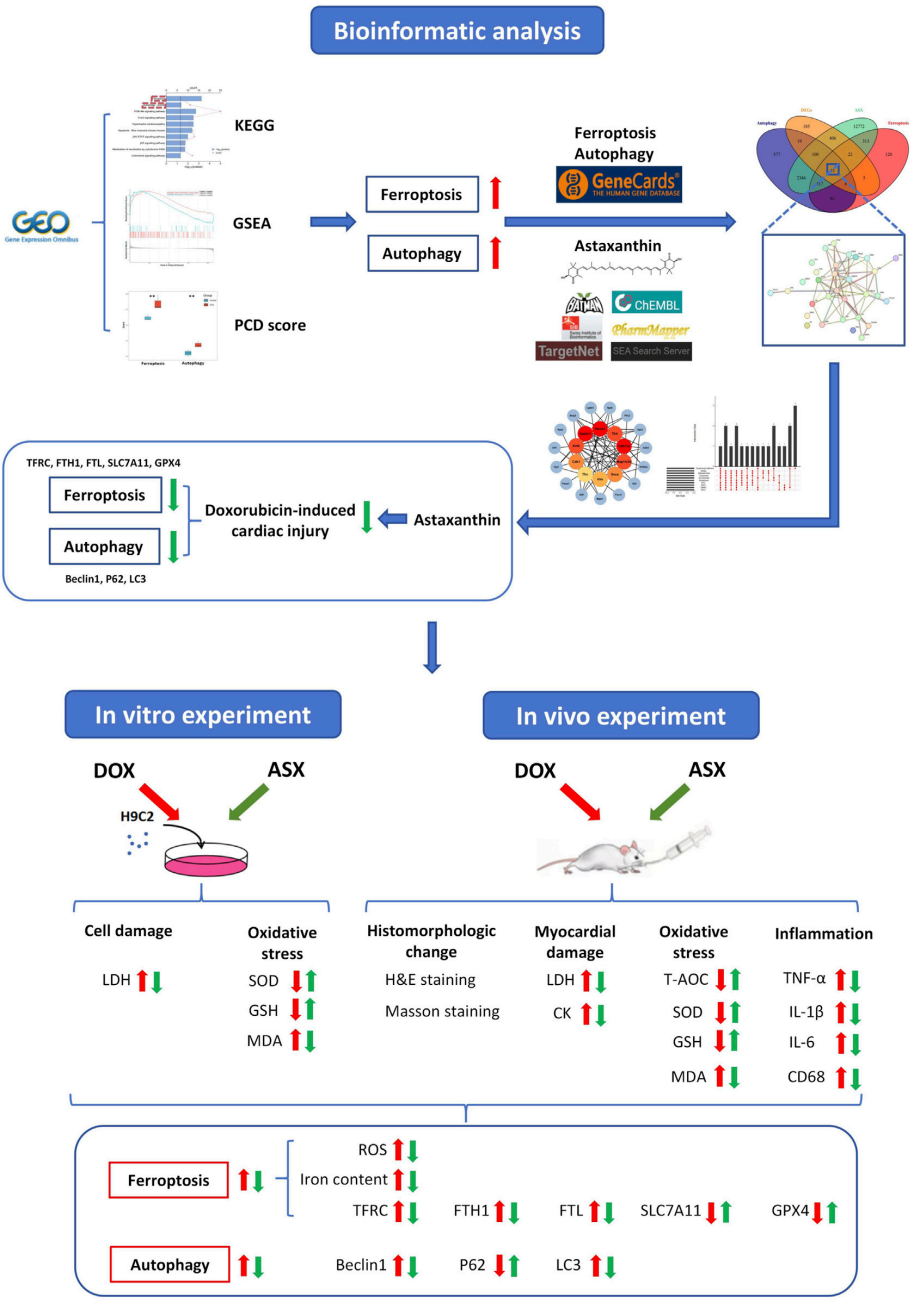
A detailed workflow of the research strategy.

Multiple previous studies have shown that natural supplements can play a good role in cardiovascular protection. Cranberry flavonoids may influence the development of atherosclerosis (Reed, 2002). Proanthocyanidins can effectively inhibit heart damage caused by PM_2.5_ (Yin et al., 2023a). Astaxanthin (ASX) is a kind of natural carotenoid with strong antioxidant ability, which is widely found in shrimp, crab, fish, birds, some algae and fungi. It has been proven that ASX has a protective effect against cardiovascular disease, respiratory disease, autoimmune disease, and cancer (Hua et al., 2023; Li et al., 2023; Yin et al., 2023b). Multiple studies have confirmed that the regulation of ferroptosis and autophagy constitutes an effective mechanism for alleviating cardiovascular injury. Cyanidin-3-glucoside and puerarin can effectively alleviate cardiac ischemia-reperfusion injury and heart failure by inhibiting ferroptosis (Liu et al., 2018; Shan et al., 2021). Ginsenoside Rg1 can attenuate alcohol-induced myocardial injury through the modulation of autophagy (Tian et al., 2023). Inhibition of autophagy by Astragaloside IV improves lipopolysaccharide-induced cardiac dysfunction (Wang et al., 2022). The findings of Wang et al. indicated that ASX can enhance cardiac function during myocardial infarction recovery by inhibiting ferroptosis (Shen et al., 2024). Our laboratory previously reported that ASX alleviated PM_2.5_-induced cardiac injury by inhibiting cardiomyocytes ferroptosis (Ren et al., 2023). Additionally, astaxanthin has been shown to exert therapeutic effects in various diseases by modulating autophagy (Kim and Kim, 2019). We hypothesize that astaxanthin may exert an anti-DIC effect by modulating ferroptosis and autophagy. This study aims to investigate the protective effects of ASX against DOX-induced cardiac injury through morphology observation and the assessment of changes in inflammation and oxidative stress levels. In addition, network pharmacology combined with *in vitro* and *in vivo* experiments will be employed to explore whether the regulation of ferroptosis and autophagy is a potential mechanism underlying the anti-DIC effects of ASX (Figure 1). This presents a novel strategy for the clinical prevention and management of DOX-induced cardiotoxicity.

## 2 Materials and methods

### 2.1 Bioinformatics analysis

We searched the Gene Expression Omnibus (GEO, https://www.ncbi.nlm.nih.gov/geo/) database, and retrieved the RNA-seq dataset GSE59672 (public on 23 Jul 2014). The gene expression profiles of three mouse after treatment with DOX (100 mg/kg) and three normal mouse were included in this dataset, and it was performed on the GPL1261. With the help of the “DEseq2” package, we selected the differentially expressed genes (DEGs), and this operation process was implemented in R software (R version 4.0.3). We set the screening condition to adjust p-value <0.05 and |log2FoldChange| ≥ 0.5. The “clusterProfiler” R package was used to conduct the Kyoto Encyclopedia of Genes and Genomes (KEGG) analyses for the DEGs. When adjust p-value <0.05, we considered the results to be statistically significant. And then we visualized it by an online platform for data analysis and visualization (https://www.bioinformatics.com.cn). GSEA 4.1.0 software was used for gene set enrichment analysis. The screening criterion was P < 0.05. We conducted a literature search (Hu et al., 2024; Qin et al., 2023) and gathered key regulatory genes of ferroptosis and autophagy, which included 87 genes related to ferroptosis and 367 genes related to autophagy. DatExpr0 () from the ‘flashClust’ package was used to remove the low-quality genes. Subsequently, the ssGSEA function in the ‘IOBR’ package was used to score the signature, the signature was set to genes associated with ferroptosis and autophagy. The minimum inclusion gene for each programmed death mode was set to 5. Ggplot () function in the “ggplot” package was used to visualize the results of the PCD score.

### 2.2 Computational pharmacology prediction

Similarity Ensemble Approach (SEA, http://sea.bkslab.org/), Swiss Target Prediction (https://swisstargetprediction.ch/), TargetNet (http://targetnet.scbdd.com/), PharmMapper (http://lilab-ecust.cn/pharmmapper/index.html), Bioinformatics Analysis Tool for Molecular mechanism of Traditional Chinese Medicine (BATMAN-TCM, http://bionet.ncpsb.org.cn/batman-tcm/), and ChEMBL (https://www.ebi.ac.uk/chembl/) were used to predict the candidate targets of ASX. Genes related ferroptosis and autophagy were obtained from GeneCards (Safran et al., 2010) (https://www.genecards.org) and OMIM (https://omim.org/).

### 2.3 Protein-protein interaction (PPI) network analysis

The obtained intersection genes was inputted into the STRING database (https://cn.string-db.org/), specifying the species as “*Mus musculus*” in order to access PPI data. The results in TSV format were exported and imported into the Cytoscape 3.9.1 software for creating visualization. Then 10 topology methods were conducted to analyze the network through CytoHubba, including ClusteringCoefficient, Stress, Betweenness, Closness, EcCentricity, BottleNeck, EPC, MNC, DMNC and MCC. The genes were sorted into descending order by their values, and the top 10 genes were regarded as the hub genes.

### 2.4 Molecular docking

The 2D structure of ASX was downloaded from PubChem (https://pubchem.ncbi.nlm.nih.gov/), and the A-fold structure of the hub target was gained from the UniProt database (https://www.uniprot.org/). The three-dimensional (3D) structure of the hub target was obtained from Protein Data Bank (PDB) database (http://www.rcsb.org/). PyMoL (version 1.7.2.1) software was used to shed excessive ligands. Docking ligands and receptors were inputted into AutoDock Tools V1.5.6 (http://autodock.scripps.edu/) software for routine pre-treatment and saved in PDBQT format. AutoDockVinaV1.1.2 was used to perform molecular docking. Finally, the 3D docking drawing was visualized by PyMOL software.

### 2.5 Materials and reagents

DOX (#23214-92-8; purity: 98%) was obtained from J&K Scientific (Beijing, China). ASX (#A9241) with a purity of ≥96% was obtained from Solarbio Science & Technology Co., Ltd. (Beijing, China). Olive oil (#8001-25-0) was obtained from Yuanye Biotechnical Company (Shanghai, China). 3-Methyladenine (3-MA; #5142-23-4; purity: 99.91%) and Ferrostatin-1 (Fer-1; #347174-05-4; purity: 99.71%) were purchased from MedChemExpress (Shanghai, China). Primary antibodies against TFRC (#A5865), FTH1 (#A19544), FTL (#A11241), SLC7A11 (#A2413), GPX4 (#A1933), GAPDH (#A19056) were obtained from ABclonal (Wuhan, China). Primary antibodies against P62 (#CY556) and Beclin1 (#CY5092) were obtained from Abways (Shanghai, China). Primary antibodies against LC3-I/II (#14600-1-AP) were obtained from Proteintech (Wuhan, China). The assay kits for malondialdehyde (MDA, #A003-1), lactate dehydrogenase (LDH, #A020-2), iron (#A039-2-1), creatine kinase (CK, #A032-1-1), glutathione (GSH, #A006-2-1) and Total antioxidant capacity (T-AOC, #A015-2-1), superoxide dismutase (SOD, #A001-3), were purchased from Nanjing Jiancheng Bioengineering Institute (Nanjing, China). Enzyme-linked immunosorbent assay (ELISA) kits for interleukin-1β (IL-1β, #ml037361), interleukin-6 (IL-6, #ml064292), tumor necrosis factor-α (TNF-α, #ml002859), and were purchased from Shanghai Enzymelinked Biotechnology Co., Ltd (Shanghai, China).

### 2.6 Animals and cells

Thirty 8-week-old male Sprague−Dawley rats, weighing 251–275 g, were provided by Beijing Vital River Laboratory Animal Technology Co., Ltd. The experimental protocols for the animals were conducted in accordance with the guidelines of Animal Experiments and were approved by the Animal Experimentation Ethics Committee of Hebei Medical University (IACUC-Hebmu-P2024055). The approval date was 9 July 2024. The animals were housed under the standard environment (24°C ± 1°C, 50% humidity, and 12 h dark/light cycle). Three rats was housed in a cage (dimensions: 485 mm × 350 mm × 200 mm) to ensure adequate living space. To maintain a comfortable living environment for the rats, the bedding was changed every 3 days. Food and water were provided regularly to ensure the rats were not deprived of nourishment or hydration.

The H9C2 cell line was obtained from Shanghai Zhong Qiao Xin Zhou Biotechnology Co., Ltd. (#ZQ0102, Shanghai, China). At 37°C with 5% CO_2_, we cultured H9C2 cells in DMEM high-glucose medium (#ZT316000-83, ZETA LIFE Inc., San Francisco, United States) supplemented with 1% penicillin and streptomycin and 10% FBS (#Z7185FBS-500, ZETA LIFE Inc., San Francisco, United States).

### 2.7 *In vitro* experimental design

Different concentrations of ASX, DOX, Fer-1 and 3-MA were used to treat H9C2 cells in order to determine the optimal intervention concentration. The effects of different concentrations of intervention were evaluated by cell viability assay. To confirm that ASX pretreatment could alleviate ferroptosis and autophagy, thereby protecting cardiomyocytes against DOX toxicity, H9C2 cells were divided into five groups: 1) control, 2) DOX, 3) ASX, 4) Fer-1, and 5) 3-MA. Prior to intervention, the cells were inoculated and cultured in medium for 12 h. H9C2 cells in the DOX group were incubated in normal medium for 24 h, followed by 12 h of exposure to 1 μM DOX. Similarly, cells in the ASX, Fer-1, and 3-MA groups were treated with 40 μM ASX, 2 μM Fer-1, and 5 mM 3-MA, respectively, for 24 h, prior to a 12-h exposure to 1 μM DOX (Figure 3B). The control group cells were incubated in normal medium for 48 h. Cell viability, iron content, SOD, GSH, LDH, and MDA were measured, along with the expression levels of TFRC, FTL, FTH1, SLC7A11, GPX4, P62, Beclin1, and LC3 in the lysate of H9C2 cells.

### 2.8 Cells cultures and treatments

The cells were exposed to different concentrations of DOX and ASX to select the optimal concentration and intervention time. Before treatment with DOX, we used ASX (40 μM), Fer-1 (2 μM) and 3-MA (5 mM) to pretreat H9C2 cells for 12 h. This protocol was designed to investigate whether ASX could mimic the roles of Fer-1 and 3-MA in attenuating DOX-induced ferroptosis and autophagy.

### 2.9 Cell viability assay

In this research, cell viability was evaluated by the Cell Counting Kit-8 (CCK-8; #K009; ZETA LIFE Inc., San Francisco, United States). All experimental procedures were conducted in accordance with the manufacturer’s instructions. PBS was used to wash the cells, after cell grouping and treatment. Subsequently, H9C2 cells were incubated at 37 °C for an additional 2 h with a mixture of 10 μL CCK-8 reagent and 100 μL fresh medium. Finally, absorbance was measured at 450 nm.

### 2.10 *In vivo* experiments design

After 1 week of adaptive feeding, rats were randomly appointed into five groups (n = 6): (i) Control group (Control), (ii) DOX group (DOX), (iii) DOX + Low-dose ASX group (DOX + ASX-L), (iv) DOX + High-dose ASX group (DOX + ASX-H), (v) High-dose ASX group (ASX-H). Based on previous studies, we designed the grouping scheme for this study (Chen et al., 2020; Qi et al., 2020). The Control group and DOX group were given daily with olive oil (5 mL/kg/day) via oral gavage for 28 days. ASX (ASX-L: 7.5 mg/kg; ASX-H: 15 mg/kg) was dissolved completely in olive oil. The dosage selection of ASX in this investigation refers to previous studies (Ren et al., 2023; Yin et al., 2023a). The solubility of low and high doses of ASX in olive oil is 1.5 and 3 mg/mL, respectively. We used a cryogenic ultrasonic water bath to assist ASX dissolution. Different concentrations of ASX were administered to DOX + ASX-L group, DOX + ASX-H group and ASX-H group via gavage every day for 28 days. DOX (2.5 mg/kg) was dissolved in normal saline to obtain a red solution. ConFigureured DOX solution was gave to DOX group, DOX + ASX-L group and DOX + ASX-H group by intraperitoneal injection on the last two experimental weeks. The exact interval of doxorubicin administration was three times per week (every other day) continued for 2 weeks. Each rat in the doxorubicin-treated groups received a cumulative dose of 15 mg/kg DOX. The administration method and dosage of DOX were based on previous studies (Sheibani et al., 2020). The other groups were given the same amount of saline via intraperitoneal injection. At the end of the last experimental week, all rats were anesthetized with pentobarbital sodium (50 mg/kg, i. p.) and executed. And then heart tissues and the serum samples of abdominal aorta were collected from the sacrificed rats. The specific experimental procedures are illustrated in Supplementary Figure S1.

### 2.11 Histopathological examination

After fixation in 4% paraformaldehyde, rat heart tissues were routinely embedded in paraffin and sectioned transversely. Place the cardiac paraffin sections on the staining rack and incubate them in an oven at 65°C for 2 h to prevent detachment. Deparaffinize the sections sequentially using xylene, ethanol, and distilled water. Dewaxed thin sections were stained with hematoxylin–eosin (H&E). After dehydration and encapsulation, slices are used to evaluate heart histological features under an optical microscope. Pathological grading of cardiac inflammation was assessed according to a previously described scoring system (Du et al., 2017), with myocarditis scores ranging from 0 to 4: 0, no inflammatory infiltrates; 1, small foci of infammatory cells between myocytes; 2, larger foci of >100 inflammatory cells; 3, >10% of a cross-section involved; 4, >30% of a cross-section involved. Additionally, tissue fibrosis was evaluated using Masson’s trichrome staining. The experimental procedure is similar to that of H&E staining. We applied color differentiation to distinguish connective tissue and recorded the percentage of the fibrotic tissue area.

Regarding immunohistochemistry (IHC), the dewaxed thin sections were treated with citrate buffer and blocked with 10% goat serum. They were then incubated overnight with the primary antibody (anti-CD68), followed by incubation with a horseradish peroxidase-conjugated secondary antibody. Subsequently, the sections were incubated overnight with the primary antibody (anti-CD68), and later treated with a horseradish peroxidase-conjugated secondary antibody. A pathologist used Image-Pro Plus 6.0 software to annotate and quantify the area of the brown immunohistochemical positive staining. Prior to analysis, a specific brown-positive region in a image was selected as the reference standard for positive identification, and the positive regions in each image were measured against this standard. The mean area percentage of positive CD68 immunoreactivity served as the final data. The experimental procedures and quantitative methods is based on previous research (Ren et al., 2024). The analysts were unclear about the content of the experiment.

### 2.12 Measurement of cardiac enzymes

A high-speed homogenizer was employed to homogenize rat heart tissues in ice-cold 0.9% normal saline (10%, w/v). The supernatant was collected after centrifugation at 3,000 rpm for 10 min at 4°C. The activities of LDH and CK were utilized to evaluate heart function. CK and LDH activities in both rat heart tissues and serum were quantified using respective determination kits. H9C2 cells were lysed with RIPA Lysis Buffer (#R0010, Solarbio Science & Technology Co., Ltd., Beijing, China) and supernatant was collected. LDH activities in the cell homogenate supernatant were assessed using colorimetric assay kits.

### 2.13 Measurement of infammatory cytokines and oxidative stress

The preparation method of tissue homogenate is the same as 2.12. According to manufacturer protocol, we quantified the contents of inflammatory cytokines (TNF-α, IL-6, and IL-1β) in serum and heart tissues using ELISA kits. The activities of SOD and T-AOC, as well as the levels of MDA and GSH in rat heart tissues and serum, were measured using assay kits (Nanjing Jiancheng Bioengineering Institute, Nanjing, China). Additionally, determination kits were also employed to measure the activities of SOD and the levels of MDA and GSH in H9C2 cells. Briefly, after mixing the sample with the enzyme solution, the mixture was incubated in a water bath at 37°C for 20 min to allow for complete reaction. Superoxide anions (O_2_−) can react with WST-1 to form a water-soluble yellow formazan, which absorbs at 450 nm. SOD scavenges O_2_−, inhibiting formazan formation. A deeper yellow color indicated lower SOD activity, while a lighter color indicated higher activity (Peskin and Winterbourn, 2000). For the detection of MDA, the sample was mixed with an equal volume of the configured working fluid and incubated in a water bath at 95°C for 40 min. The supernatant was collected after centrifugation at 3,500 rpm for 10 min at 4°C. MDA can react with thiobarbituric acid (TBA) under high temperature and acidic conditions to form a red product, with a peak absorption at 532 nm (Janero, 1990). The detection processes for GSH and T-AOC are largely similar. The reagents provided in the kit were thoroughly mixed with the sample, followed by incubation at room temperature for 5 min. GSH can react with 5,5′-dithiobis (2-nitrobenzoic acid) (DTNB) to form a yellow compound, which can be quantitatively measured at 405 nm to determine GSH content (Smith et al., 1988). ABTS (2,2′-azino-bis(3-ethylbenzothiazoline-6-sulfonic acid)) was oxidized to the green ABTS^+^ by an appropriate oxidant. The production of ABTS^+^ was inhibited in the presence of antioxidants. The T-AOC of the sample can be calculated by measuring the absorbance of ABTS^+^ at 734 nm or 405 nm (Erel, 2004). The detailed experimental procedures are provided in the instructions (http://www.njjcbio.com/).

### 2.14 Flow cytometric analysis of ROS

The generation of reactive oxygen species (ROS) in heart tissues was determined using a ROS assay kit (#Ros100, ZETA LIFE Inc., San Francisco, United States) containing dichlorofluorescein diacetate (DCFH-DA). Freshly isolated rat heart were cut with tissue shears and then digested with trypsin for 20 min. Upon completion of digestion, the precipitate was filtered through a 70 μm cell filter, followed by centrifugation at 1,200 rpm for 10 min to collect the precipitate. Subsequently, the ROS fluorescent probe was added, and the mixture was incubated at 37°C for 30 min in a constant-temperature incubator. After incubation, the cells were centrifuged at 1,000 rpm for 10 min and the cell precipitate was collected. The cells were then washed 3 times with PBS. Finally, the precipitate was added with appropriate amount of PBS and detected by flow cytometry.

### 2.15 Iron content detection

The commercially available colorimetric assay kit (#A039-2-1, Nanjing Jiancheng Bioengineering Institute, Nanjing, China) were utilized in strict accordance with the kit instructions, to determine the iron content in the H9C2 cell and rat myocardium. Briefly, we used a microplate reader to measure the optical density (OD) value at 520 nm and determined the iron content in the samples by comparing the OD of the samples to the standard curve.

### 2.16 Western blot

Total proteins from the lysates of H9C2 cells and myocardium were extracted using the RIPA buffer containing protease inhibitors. The BCA Assay Kit (#A045-4-2, Nanjing Jiancheng Bioengineering Institute, Nanjing, China) was used to quantify protein concentrations. Protein samples were separated through 8%–12% sodium dodecyl sulfate-polyacrylamide gelelectrophoresis (SDS-PAGE) and transferred onto polyvinylidene fluoride (PVDF) membranes. The membrane was blocked with 5% skimmed milk at 25°C for 2 h. According to the location of the target protein bands, the membrane was cut into small strips. After washed by Tris buffered saline Tween (TBST) three times (10 min each), all cropped membranes were incubated overnight at 4°C with different specific primary antibodies. At the end of incubation, membranes were cleaned three times with TBST for 10 min each time. Then, the membranes were incubated for 40 min at 25°C with an HRP-conjugated secondary antibody (#AB0103, Abways, Shanghai, China). Subsequently, the bands were washed again with TBST three times, and imaged by an enhanced chemiluminescence detection system. The gray density quantification was performed by ImageJ analysis software 1.8.0. GAPDH was used as an internal control, to normalize expression levels.

### 2.17 Statistical analyses

SPSS 21.0 and GraphPad Prism software 6.0 was used to analyze the data and display the results. All numerical data were shown as the mean ± standard error of the mean (SEM). Shapiro-Wilk test was used to check the normality of the data. The differences among multiple groups was calculated by one-way ANOVA analysis followed by Dunnett’s test. If the data did not satisfy the assumptions of normality, nonparametric tests (Kruskal-Wallis H test) were utilized. A difference was considered statistically significant when the *p*-value was less than 0.05. Every possible comparison between the study groups was considered.

## 3 Results

### 3.1 Network pharmacology prediction

**FIGURE 2:**
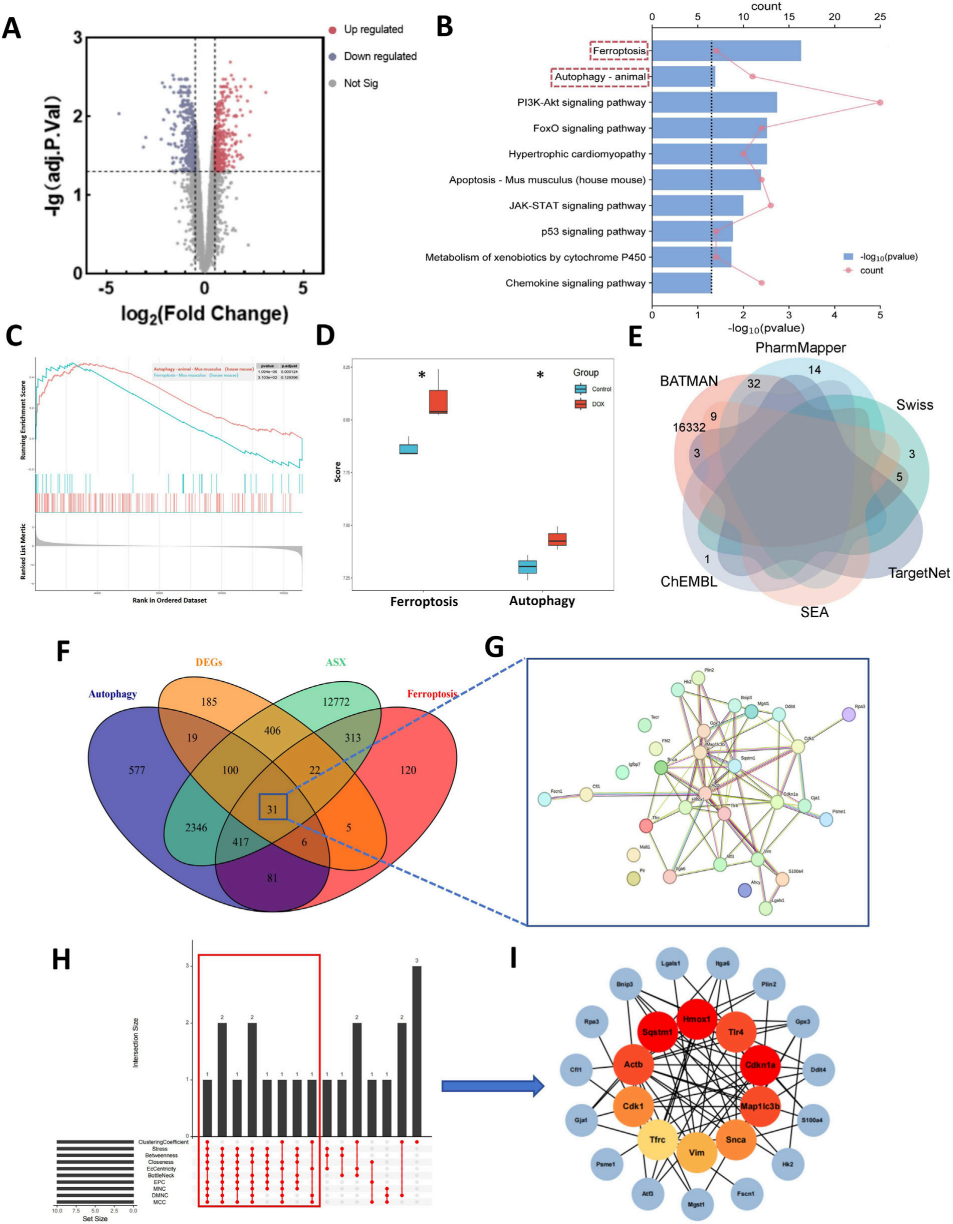
Computational pharmacology analysis of doxorubicin (DOX) and astaxanthin (ASX). **(A)** The differentially expressed genes (DEGs) between normal mouse heart tissue and heart tissue after treatment with DOX. **(B)** Kyoto Encyclopedia of Genes and Genomes (KEGG) enrichment analysis of DEGs. **(C)** Results of Gene Set Enrichment Analysis (GSEA) of Ferroptosis and Autophagy. **(D)** Gene Set Variation Analysis (GSVA) of 2 PCD pathways (ferroptosis and autophagy) demonstrated by box plots in Control and DOX groups. **(E)** Venn graph showing the numbers of predicted ASX targets. **(F)** The overlapping targets of ASX, DEGs, Ferroptosis and Autophagy. **(G)** A total of 31 interaction targets were used for PPI network visualization. **(H)** UpSet plot of 10 topological algorithms. The core target is shown in the red box. **(I)** The PPI network of overlapping targets. The red nodes represent the core targets selected by the topological algorithms.

Compared to normal mice, a total of 774 DEGs were identified in the mice treated with DOX, comprising 385 upregulated and 389 downregulated genes (Figure 2A). The biological function of the DEGs was identifed by KEGG pathway enrichment analyses. As illustrated in Figure 2B, the DEGs were closely associated with ferroptosis and autophagy signaling pathway. Results of GSEA shown that the expression of ferroptosis and autophagy pathways was significantly upregulated (Figure 2C), and they are the only two modes of cell death among all the significantly enriched pathways (Supplementary Table S1). To gain further insight into the activation status of ferroptosis and autophagy in DIC, we calculated the enrichment scores for ferroptosis and autophagy. Figure 2D revealed that ferroptosis and autophagy were upregulated in DOX group.

From the ChEMBL, PharmMapper, Swiss Target Prediction, TargetNet, and BATMAN-TCM databases, we obtained 16,407 target genes for ASX after removing partially overlapping targets (Figure 2E). Using “ferroptosis” and “autophagy” as keywords, we identified 995 and 3,577 genes on GeneCards and OMIM, respectively. As shown in Figure 2F, there were 31 target genes overlapped among the target genes of ASX, DEGs, ferroptosis, and autophagy. A PPI network was established to search the interactions of these target genes through the STRING database. Cytoscape was used to further visualize the data (Figure 2G). After constructing the PPI network, the significance of potential therapeutic targets within the interacting network was further assessed using CytoHubba in Cytoscape, employing algorithms including CytoHubba, including ClusteringCoefficient, Stress, Betweenness, Closness, EcCentricity, BottleNeck, EPC, MNC, DMNC and MCC. The targets were evaluated and ranked, with the top 10 of targets based on their algorithm scores undergoing an intersection process. An UpSet diagram was utilized to show the number of genes. As shown in Figure 2H, a total of 10 genes were obtained from the intersection of 10 algorithms, including Cdkn1a, Hmox1, Sqstm1, Actb, Map1lc3b, Tlr4, Cdk1, Snca, Vim, Tfrc. As showcased in Figure 2I, there are multiple associations between hub genes and other target genes. Molecular Docking Analysis was used to confirm whether the bioactive components of ASX could directly interact with the hub genes. As showed in Table 1, the binding activity between ASX and hub genes was relatively good. The binding affinity lower than −5.0 kcal/mol indicates that the active ingredient has a good binding ability to protein.

**TABLE 1:** The docking result analysis of ASX and hub targets.

Target protein	Binding energy (kcal/mol)	No of H bonds	Amino acid residues forming H-bond with their length in A
Cdkn1a	−8.51	3	GLY-29 (1.9), SER-31 (2.4), SER-31 (2.0)
Hmox1	−7.6	1	SER-159 (3.4)
Sqstm1	−8.26	1	GLY-1 (1.8)
Actb	−8.08	2	Y115 (2.4), K111 (2.3)
Map1lc3b	−7.5	1	ASN-236 (2.8)
Tlr4	−7.8	3	THR-497 (3.0), SER-332 (3.1), GLN-331 (2.6)
Cdk1	−7.73	2	ARG-36 (3.2), GLU-38 (1.7)
Snca	−8.43	2	GLN-336 (1.8), LIE-330 (3.0)
Vim	−8.11	1	TRY-76 (3.0)
Tfrc	−8.14	1	PHE-299 (2.1)

Among the predicted targets, Tfrc (TFRC), Sqstm1 (P62), and Map1lc3b (LC3) are core genes in the ferroptosis and autophagy pathways. We predicted that ASX could alleviate the ferroptosis and autophagy caused by DOX. Transferrin receptor protein 1 (TFRC) is one of the key components that mediates the entry of iron into cells. The over expression of TFRC can intensify the iron uptake in cells, and promote the upregulation of ferritin components FTH1 and FTL to store iron. The enhanced iron uptake and storage ultimately lead to the accumulation of iron within the cells (Chen et al., 2021). The downregulation of key components of the intracellular antioxidant system, SLC7A11 and GPX4, leads to the accumulation of intracellular ROS (Lee et al., 2020). The combined effect of excess iron and ROS can ultimately lead to the occurrence of ferroptosis. LC3 is a key protein marker of autophagosomes, which connects to ubiquitinated proteins via P62. Together, they play a critical role in the formation of autophagic vesicles. The accumulation of LC3 and the depletion of P62 suggest the activation of autophagy (Festa et al., 2024). Beclin1 is an autophagy regulatory protein involved in the formation and degradation of autophagosomes. Over expression of Beclin1 can mediate the initiation of autophagy (Vélez et al., 2024). Based on previous relevant studies, we speculate that ASX may affect the core genes of ferroptosish signaling pathway (TFRC, SLC7A11, GPX4, FTL, and FTH1) and autophagy signaling pathway (Beclin1, P62, and LC3).

### 3.2 ASX protected against DOX-induced cell injury in H9C2 cells

**FIGURE 3:**
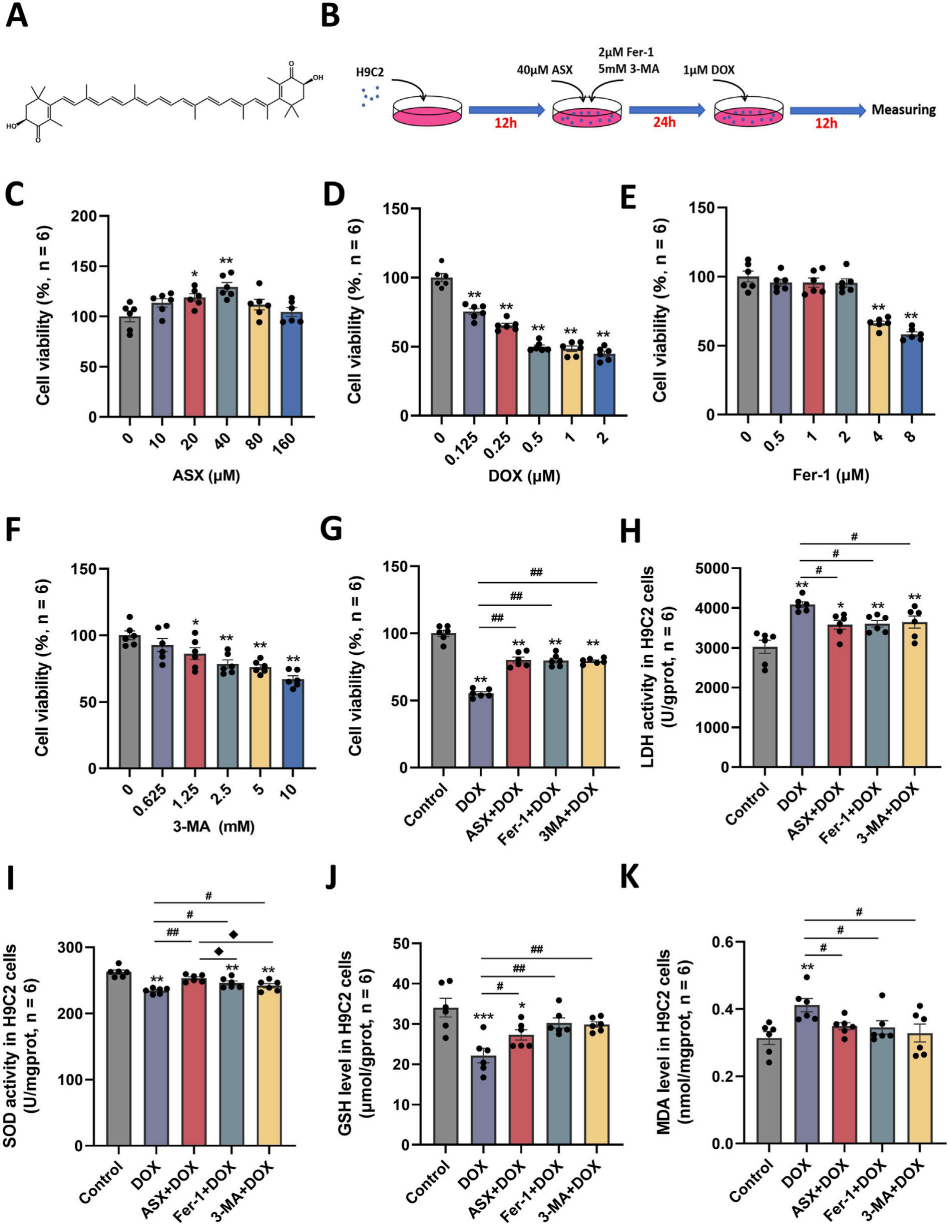
Astaxanthin (ASX) attenuates doxorubicin (DOX)-induced H9C2 cells injury. **(A)** ASX molecular formula. **(B)** Diagram of the *in vitro* study design. **(C–F)** H9C2 cells were treated with diferent concentrations of ASX, DOX, Fer-1 and 3-MA for 24 h. The cell viability was detected by CCK8 assay. **(G)** Cell viability of H9C2 cells treated with DOX, ASX, Fer-1 and 3-MA. **(H–I)** The activities of LDH and SOD activity in the H9C2 cells treated with DOX, ASX, Fer-1 and 3-MA. **(J–K)** Levels of GSH and MDA in the H9C2 cells treated with DOX, ASX, Fer-1 and 3-MA. Values are expressed as mean ± SEM (n = 6). *p < 0.05 difference from control group; **p < 0.01 difference from the Control group; #p < 0.05 difference from the DOX exposure group; ##p < 0.01 difference from the DOX exposure group; ◆p < 0.05 difference from the ASX + DOX group; ◆◆p < 0.01 difference from the ASX + DOX group; ▲ p < 0.05 difference from the Fer-1+DOX group; ▲▲p < 0.01 difference from the Fer-1+DOX group.

The molecular structure of ASX was shown in Figure 3A. To determine the optimal intervention concentrations of ASX, DOX, Fer-1, and 3-MA, we utilized various concentrations: ASX (0, 10, 20, 40, 80, 160 µM), DOX (0, 0.125, 0.25, 0.5, 1, 2 µM), Fer-1 (0, 0.5, 1, 2, 4, 8 µM), and 3-MA (0, 0.625, 1.25, 2.5, 5, 10 mM) for 24 h (Figures 3C–F). We detected cell viability by CCK-8 assay, and selected the optimal concentrations of ASX (40 µM), DOX (0.5 µM), Fer-1 (2 µM) and 3-MA (5 mM). Following treatment with ASX, Fer-1, and 3-MA, we observed a significant protective effect on the proliferative ability of H9C2 cells (Figure 3G). As depicted in Figures 3H–K, interventions with ASX, Fer-1, and 3-MA increased the activity of SOD and the levels of GSH, while simultaneously inhibiting LDH activity and the levels of MDA.

### 3.3 ASX reduced DOX-induced myocardial injury in rats

**FIGURE 4:**
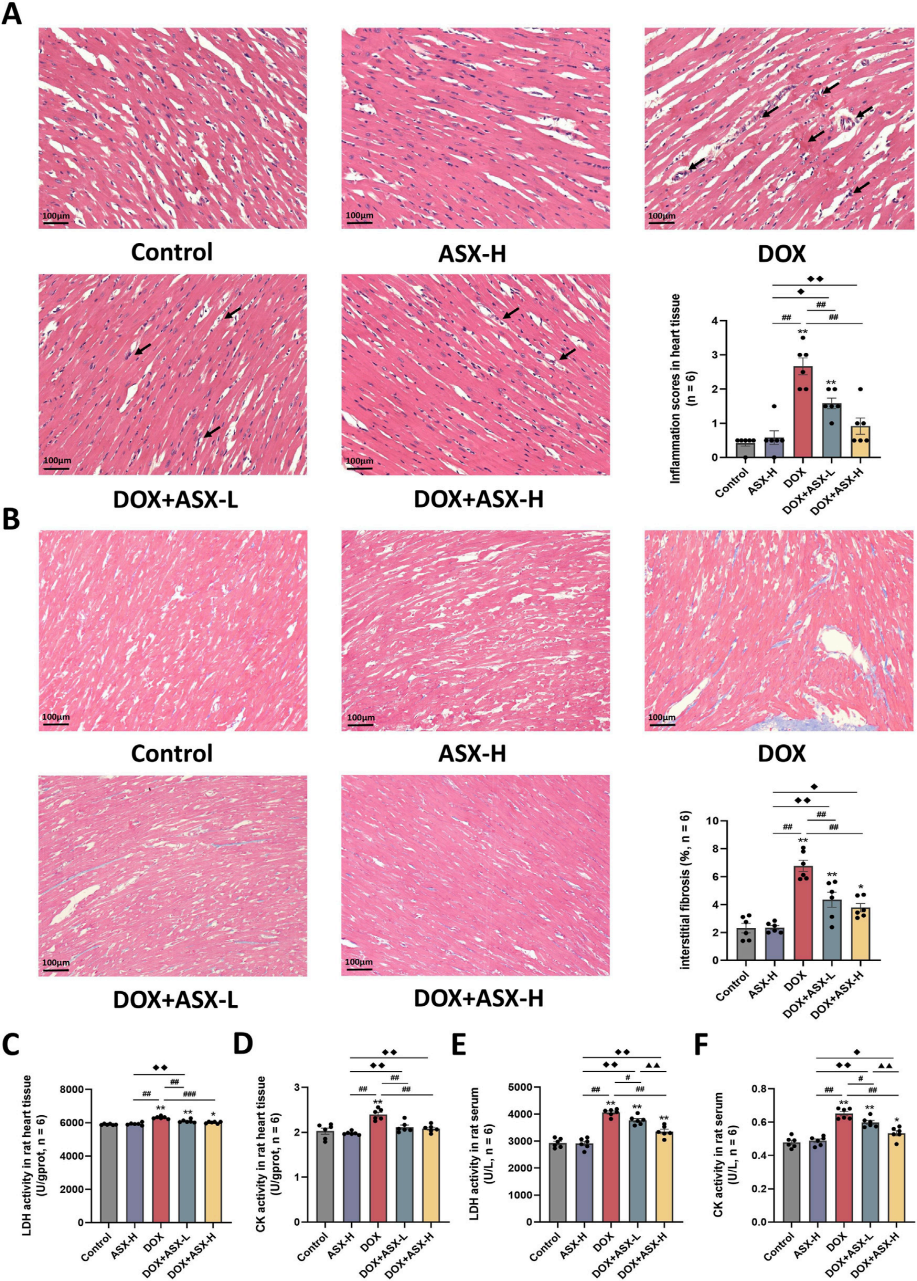
Astaxanthin (ASX) alleviates heart injury induced by doxorubicin (DOX) in rat. **(A)** Hematoxylin and eosin (H&E) staining of heart sections (magnification 200×; scale bar = 100 μm; hemorrhage, red arrow; inflammatory cell infiltration, black arrow), and the myocarditis score of heart in each group. **(B)** Masson staining of heart sections (magnification = 200×, scale bar = 100 μm) and the quantitative analyses. **(C, D)** The activities of LDH and CK activity in heart tissue. **(E, F)** The activities of LDH and CK activity in serum. Values are expressed as mean ± SEM (n = 6). *p < 0.05 difference from control group; **p < 0.01 difference from the Control group; #p < 0.05 difference from the DOX exposure group; ##p < 0.01 difference from the DOX exposure group; ◆p < 0.05 difference from the ASX-H group; ◆◆p < 0.01 difference from the ASX-H group; ▲ p < 0.05 difference from the DOX + ASX-L group; ▲▲p < 0.01 difference from the DOX + ASX-L group.

In this study, we randomly selected three mice from each group to obtain heart tissue samples. One pathological section was prepared using heart tissue samples from each animal. Two non-overlapping images were randomly captured from each tissue section. A total of six images from each group were used for the final pathological evaluation. H&E staining results demonstrated that ASX could attenuate congestion and inflammatory cell infiltration. Additionally, the heart histopathological score results indicated that the score for the DOX group was significantly increased, while ASX intervention significantly reduced the histopathological score of heart tissue (Figure 4A). Furthermore, Masson’s trichrome staining was conducted to evaluate the degree of cardiac fibrosis. As shown in Figure 4B, compared with the control group and the ASX-H group, cardiac fibrosis in the DOX group was significantly aggravated. However, cardiac fibrosis was notably alleviated after ASX intervention. Moreover, ASX also prevented cardiac function damage caused by DOX and inhibited the expression of LDH and CK in serum and heart tissue (Figures 4C–F).

### 3.4 ASX attenuated DOX-induced inflammation in rats heart

**FIGURE 5:**
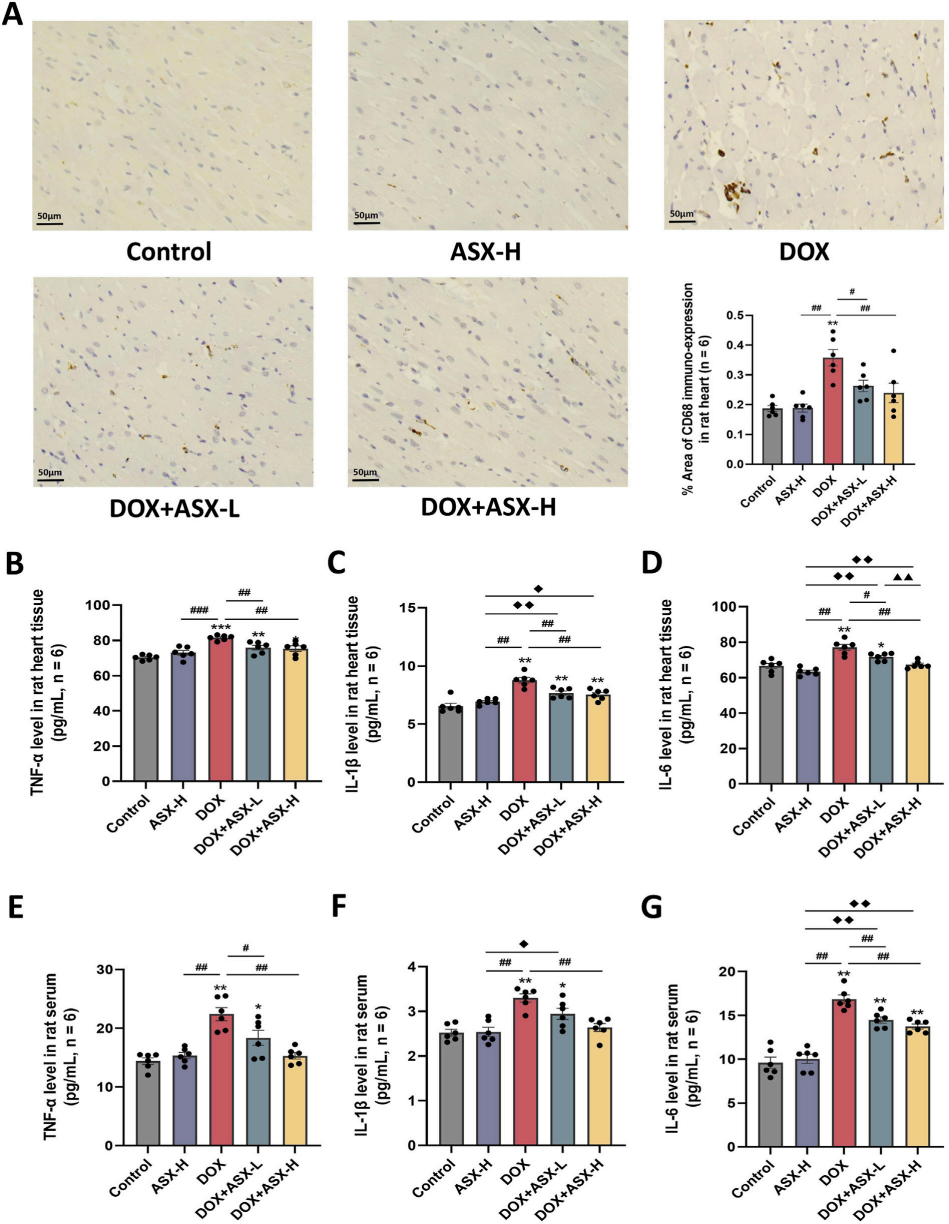
Astaxanthin (ASX) attenuates doxorubicin (DOX)-induced inflammation in heart. **(A)** Immunohistochemical staining for CD68 of heart sections (magnification = 400, scale bar = 50 μm) and the quantitative analyses. **(B–G)** TNF-α, IL-1β, and IL-6 levels in the rat heart tissue and serum. Values are expressed as mean ± SEM (n = 6). *p < 0.05 diference from control group; **p < 0.01 difference from the Control group; #p < 0.05 difference from the DOX exposure group; ##p < 0.01 difference from the DOX exposure group; ◆p < 0.05 difference from the ASX-H group; ◆◆p < 0.01 difference from the ASX-H group; ▲ p < 0.05 difference from the DOX + ASX-L group; ▲▲p < 0.01 difference from the DOX + ASX-L group.

Inflammatory response is closely related to macrophage activation, often accompanied by the infiltration of numerous macrophages. We assessed the infiltration of CD68-positive macrophages in heart tissue using immunohistochemical staining. The results showed that the percentage area of CD68-positive immuno-expression in the DOX group was markedly increased compared to the control group, with significant differences (P < 0.05). However, after ASX intervention, these positive areas were significantly reduced (Figure 5A). Additionally, to evaluate the anti-inflammatory effects of ASX in rat heart tissue, we measured the levels of TNF-α, IL-1β, and IL-6 in heart tissues and serum. As shown in Figures 5B–G, the levels of these three inflammatory cytokines were significantly elevated in heart tissues and serum after exposure to DOX. However, this upregulation was relieved by ASX pre-treatment.

### 3.5 ASX suppressed DOX-induced oxidative stress in rats heart

**FIGURE 6:**
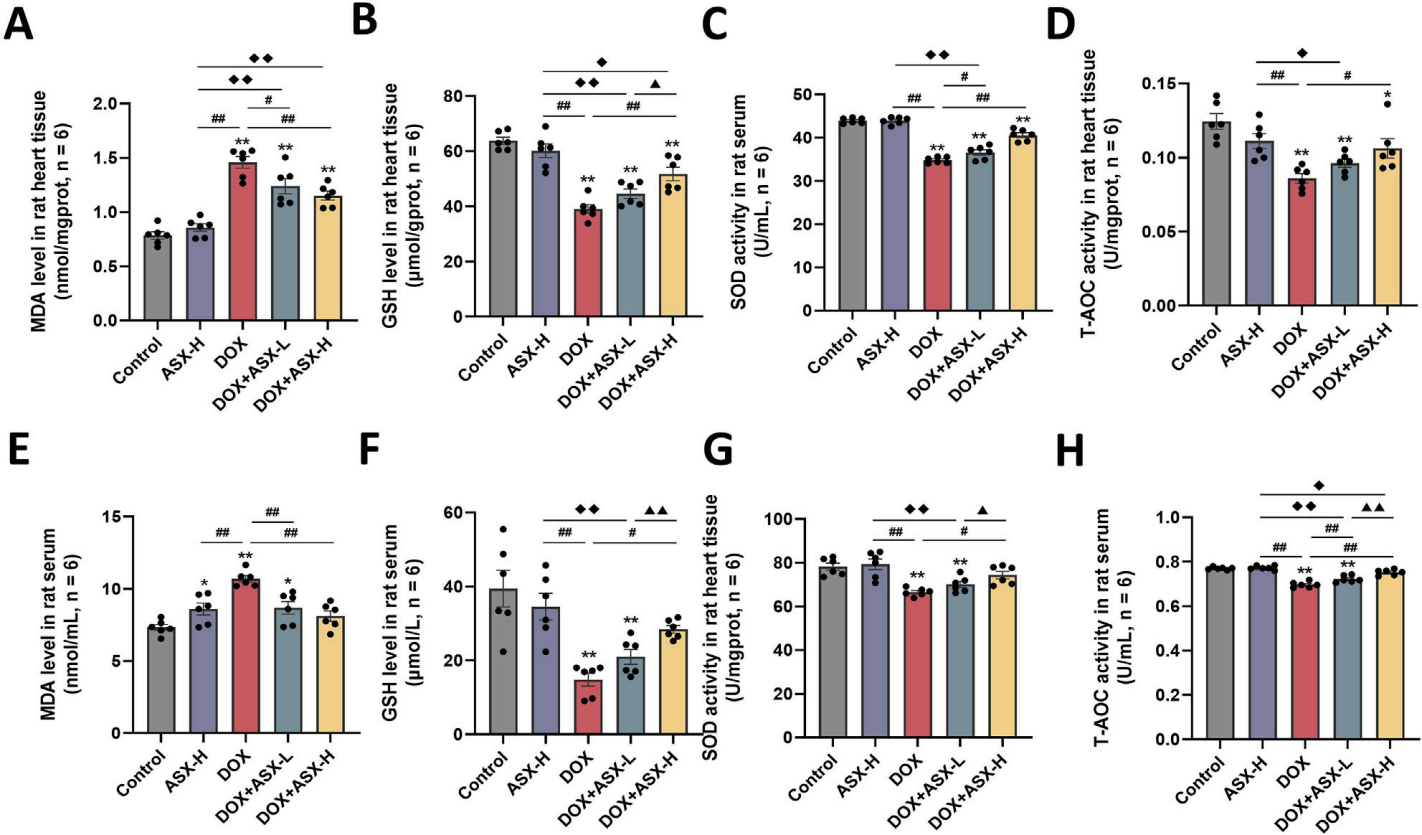
Astaxanthin (ASX) attenuates doxorubicin (DOX)-induced oxidative stress in heart. **(A, B)** Levels of MDA and GSH in the heart tissue. **(C, D)** The activities of SOD, T-AOC in the heart tissue. **(E, F)** Levels of MDA and GSH in serum. **(G, H)** The activities of SOD, T-AOC in serum. Values are expressed as mean ± SEM (n = 6). *p < 0.05 difference from control group; **p < 0.01 difference from the Control group; #p < 0.05 difference from the DOX exposure group; ##p < 0.01 difference from the DOX exposure group; ◆p < 0.05 difference from the ASX-H group; ◆◆p < 0.01 difference from the ASX-H group; ▲ p < 0.05 difference from the DOX + ASX-L group; ▲▲p < 0.01 difference from the DOX + ASX-L group.

Oxidative stress is a critical factor in DOX-induced cardiac injury. To assess the extent of cardiac damage caused by DOX exposure, we measured oxidative stress levels in cardiac tissue and serum. As shown in Figure 6, DOX exposure increased the level of MDA and inhibited the expression of several antioxidant markers, including SOD, GSH, and T-AOC in heart tissue and serum. ASX preprocessing effectively relieved the upregulation of MDA level and the downregulation of the activity of SOD, GSH and T-AOC. Therefore, ASX can alleviate oxidative stress in DOX-induced cardiac damage.

### 3.6 ASX diminished DOX-induced ferroptosis and autophagy in H9C2 cells

**FIGURE 7:**
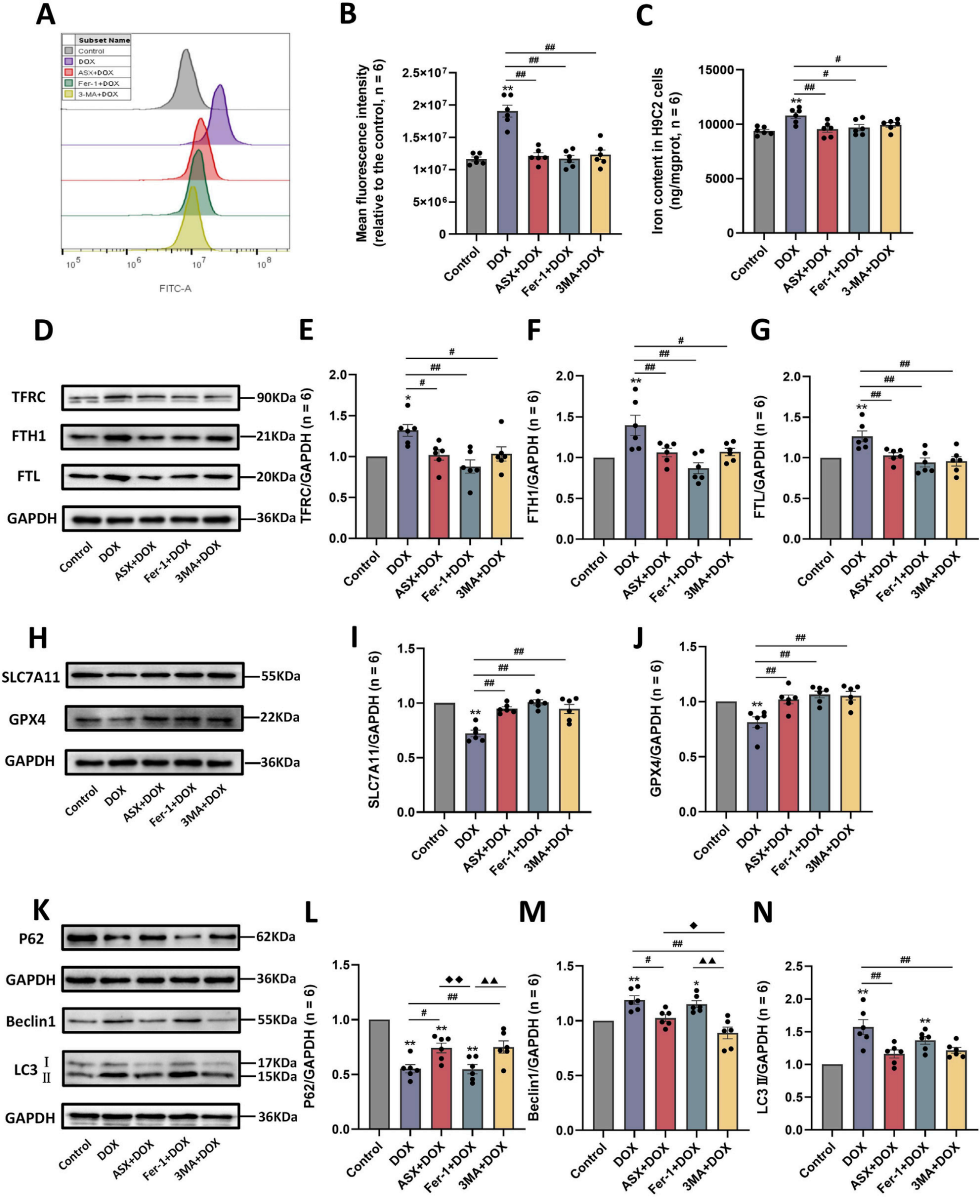
Astaxanthin (ASX) attenuates doxorubicin (DOX)-induced ferroptosis and autophagy in H9C2 cells. **(A)** Representative fluorescence intensity images of ROS by DCFH-DA obtained by flow cytometry. **(B)** Flow cytometric analysis of fluorescence intensity. **(C)** Iron content were detected in the H9C2 cells. **(D–J)** Western blotting results and quantitative analysis of ferroptosis-related proteins, including TFRC, FTH1, FTL, SLC7A11, and GPX4. **(K–N)** Western blotting results and quantitative analysis of autophagy-related proteins, including P62, Beclin1 and LC3. Values are expressed as mean ± SEM (n = 6). *p < 0.05 difference from control group; **p < 0.01 difference from the Control group; #p < 0.05 difference from the DOX exposure group; ##p < 0.01 difference from the DOX exposure group; ◆p < 0.05 difference from the ASX + DOX group; ◆◆p < 0.01 difference from the ASX + DOX group; ▲ p < 0.05 difference from the Fer-1+DOX group; ▲▲p < 0.01 difference from the Fer-1+DOX group.

To further verify whether inhibition of ferroptosis and autophagy is a potential mechanism by which ASX protects against DOX-induced cardiovascular injury, we examined reactive oxygen species (ROS) and iron accumulation, as well as the expression levels of ferroptosis-related proteins (TFRC, FTH1, FTL, SLC7A11, and GPX4) and autophagy-related proteins (P62, Beclin1, and LC3). Our results showed that DOX exposure led to excessive accumulation of ROS and iron in H9C2 cells. Compared to the DOX exposure group, the levels of iron and ROS in the ASX pre-treatment group were significantly reduced, with effects similar to those in the Fer-1 pre-treatment group (Figures 7A–C). At the same time, after exposure of H9C2 cells to DOX, the expressions of TFRC, FTH1 and FTL were significantly upregulated, while the expressions of SLC7A11 and GPX4 were significantly downregulated. ASX and Fer-1 preconditioning effectively alleviated these changes in ferroptosis-related protein expression induced by DOX in H9C2 cells (Figures 7D–J). Interestingly, 3-MA also exerted a similar effect to ASX and Fer-1, playing an inhibitory role in ferroptosis caused by DOX in H9C2 cells. In addition, we found that autophagy levels in H9C2 cells increased significantly after DOX exposure. The expression of P62 was significantly downregulated, while Beclin1 and LC3 levels were significantly upregulated. In the ASX and 3-MA intervention groups, changes in autophagy related proteins were restored. However, Fer-1 preconditioning showed no effect on DOX-induced autophagy (Figures 7K–N). These results suggest that ASX can effectively alleviate ferroptosis and autophagy induced by DOX in H9C2 cells.

### 3.7 ASX suppressed DOX-induced ferroptosis and autophagy in rats heart

**FIGURE 8:**
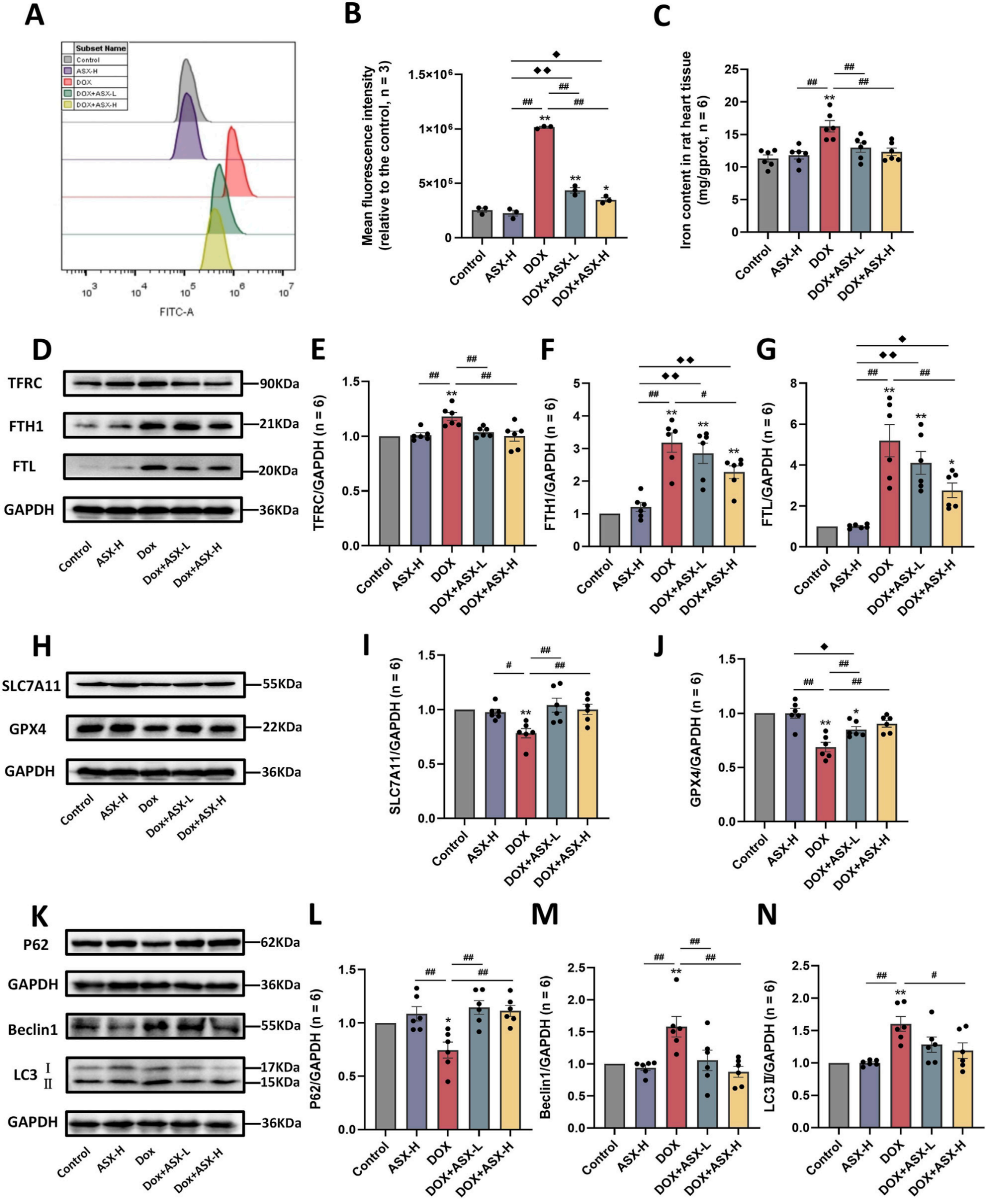
Astaxanthin (ASX) attenuates doxorubicin (DOX)-induced ferroptosis and autophagy in heart. **(A)** Representative fuorescence intensity images of ROS by DCFH-DA obtained by fow cytometry. **(B)** Flow cytometric analysis of fuorescence intensity. **(C)** Iron content were detected in the heart tissue. **(D–J)** Western blotting results and quantitative analysis of ferroptosis-related proteins, including TFRC, FTH1, FTL, SLC7A11, and GPX4. **(K–N)** Western blotting results and quantitative analysis of autophagy-related proteins, including P62, Beclin1 and LC3. Values are expressed as mean ± SEM (n = 6). *p < 0.05 difference from control group; **p < 0.01 diference from the Control group; #p < 0.05 difference from the DOX exposure group; ##p < 0.01 difference from the DOX exposure group; ◆p < 0.05 difference from the ASX-H group; ◆◆p < 0.01 difference from the ASX-H group; ▲ p < 0.05 difference from the DOX + ASX-L group; ▲▲p < 0.01 difference from the DOX + ASX-L group.

In order to determine whether ASX can alleviate ferroptosis and autophagy caused by DOX, we also conducted *in vivo* experiments. The results were consistent with those observed *in vitro*. DOX exposure caused the accumulation of ROS and iron in rat heart tissue (Figures 8A–C). Meanwhile, the expression levels of ferroptosis-related proteins TFRC, FTH1, and FTL were significantly increased, while the expressions of SLC7A11 and GPX4 were significantly decreased (Figures 8D–J). ASX pretreatment at different concentrations effectively mitigated the changes in a series of ferroptosis-related indicators caused by DOX, with the high-dose intervention group showing particularly pronounced effects (Figures 8A–J). Regarding autophagy, compared to the control and ASX-H groups, the expression level of P62 in the DOX group was significantly decreased, whereas the levels of Beclin1 and LC3 were significantly increased. However, in the ASX intervention group, the upregulation of Beclin1 and LC3 and the downregulation of P62 were alleviated to some extent (Figures 8K–N).

## 4 Discussion

With societal development and changes in people’s living habits, the incidence of malignant tumors is increasing. Chemotherapy-related side effects increasingly affect the quality of life of cancer patients, particularly cardiac toxicity (Fitzmaurice et al., 2015; Miller et al., 2019). Cardiac damage caused by DOX has become a hot topic in cardiovascular research in recent years, and a large number of experimental studies have explored anti-DIC drugs according to the characteristics of DIC. The mechanism of doxorubicin-induced cardiotoxicity is complex and may involve multiple forms of cell death (Christidi and Brunham, 2021; Galluzzi et al., 2018; Tang et al., 2019). However, it also presents more opportunities for identifying intervention targets. A large number of studies have shown that ferroptosis and autophagy are closely related to doxorubicin-induced heart damage (He et al., 2021; Lin et al., 2024; Ou et al., 2024; Wang et al., 2024; Zhai et al., 2024). Overlapping and crosstalk among two types of programmed cell death may the potential mechanism of DIC. Dexrazoxane primarily acts as an iron chelator to inhibit ferroptosis during DIC, which may be crucial for its anti-DIC effect, as suggested by Stoyanovsky et al. (2019). Elucidating this mechanism is essential for the development of targeted inhibitors. Some drugs that may inhibit the overproduction of ROS do not play an effective anti-DIC role, indicating that simple antioxidant and inhibition of ROS production are not enough to fully control DIC, which further proves the complexity of DIC mechanism and the necessity of elucidating its mechanism (Galluzzi et al., 2018; Takemura and Fujiwara, 2007; Varela-López et al., 2019).

Currently, many natural compounds have been shown to effectively ameliorate DOX-induced cardiotoxicity by modulating multiple molecular pathways. For instance, fisetin can alleviate DIC by activating the SIRT1/Nrf2 signaling pathway and inhibiting ferroptosis (Li et al., 2022). Berberine regulates autophagy and inhibits apoptosis, thereby protecting cardiomyocytes from DIC (Coelho et al., 2017; Wu et al., 2019). ASX is a widely used natural compound known for its powerful antioxidant and anti-inflammatory properties. Previous studies have shown that ASX can intervene in various forms of regulated cell death, including autophagy, apoptosis, and ferroptosis (Cai et al., 2022; Ma H. et al., 2020). In our study, a series of morphological, functional, and enzymatic indicators suggested that ASX can effectively relieve DIC. The data showed that ASX significantly attenuated oxidative stress and inflammatory damage due to DOX exposure *in vivo* and *in vitro*. In addition, ferroptosis inhibitor Fer-1 and autophagy inhibitor 3-MA was used to further investigate the underlying mechanism. The study results indicated that ASX pretreatment could markedly protect cardiomyocytes from DOX-induced ferroptosis and autophagy *in vitro* and *in vivo*. This is consistent with the function of Fer-1 against ferroptosis and 3-MA against autophagy.

As a novel research method, network pharmacology has been widely used in studying disease signaling disturbances and drug modes of action in recent years. In this study, 774 DEGs were obtained from the GEO database. These DEGs can regulate multiple signaling pathways and may contribute to the development of DIC. The results of KEGG enrichment analysis showed that the DEGs are mainly related to ferroptosis, autophagy, hypertrophic cardiomyopathy, apoptosis, etc. This suggests that ferroptosis and autophagy are critical in DOX-induced cardiac damage and is consistent with current experimental findings (Chen et al., 2022; He et al., 2021). The result of GSEA and enrichment scores shown that the expressions of ferroptosis and autophagy pathway were significantly upregulated in DOX group. The enhancement of ferroptosis and autophagy may be the key causes of cardiac cell death and cardiac dysfunction caused by DOX. Next, Venn diagram of ASX targets, DEGs, and the genes associated with ferroptosis and autophagy was used to explore the possible therapeutic targets for ASX. Then, based on 31 overlapping genes, we built the PPI network with the STRING database to identify the core genes. Docking analysis further verifed that ferroptosis and autophagy related proteins (Tfrc, Sqstm1, Map1lc3b, Cdkn1a, Hmox1, Actb, Tlr4, Cdk1, Snca, Vim) might be the potential targets for the prevention of DIC with ASX. Tfrc (TFRC), Sqstm1 (P62), and Map1lc3b (LC3) are core genes in the ferroptosis and autophagy pathways. The results of network pharmacology demonstrated that the regulation of ferroptosis and autophagy may be potential mechanisms through which ASX alleviates DIC, but further experimental verification is still needed.

In previous experimental studies, DIC often showed morphological changes, disturbance of oxidation balance, aggravation of inflammation and other damage manifestations (Guo et al., 2016; Radeva-Ilieva et al., 2020). Therefore, we also detected morphological, oxidative stress and inflammation indicators to measure the degree of damage caused by DOX. In this study, we investigated the anti-DIC effects of ASX using both *in vivo* and *in vitro* models. Our results demonstrate that DOX induces alterations in a series of oxidative stress markers, such as SOD, GSH, MDA, and T-AOC in rat heart tissue and serum. DOX also enhances macrophage infiltration and increases the expression of inflammatory factors, including TNF-α, IL-6, and IL-1β, consistent with findings from previous studies (Ammar et al., 2012). ASX effectively mitigates DOX-induced morphological, oxidative stress, and inflammatory damage in rat heart tissue. Moreover, ASX alleviates the decreased viability and oxidative damage in H9C2 cells caused by DOX. The ASX intervention group’s performance closely resembled that of the control group, indicating that ASX is safe and non-toxic at the intervention concentrations used in our study (Jiang et al., 2020). These findings suggest that ASX can effectively mitigate DOX-induced heart damage, but the specific regulatory mechanisms require further experimental verification.

*In vitro* experiments revealed that Fer-1 and 3-MA exhibit protective effects similar to those of ASX against various injuries caused by DIC. Fer-1 and 3-MA can alleviate the decrease in H9C2 cell viability and the increase in oxidative stress induced by DOX to a certain extent. Similar results have been shown in several studies (He et al., 2021; Ren et al., 2023). This suggests that inhibiting ferroptosis and autophagy may be a viable strategy for alleviating DIC. Further results showed that DOX indeed induces ROS production and iron accumulation, as well as changes in the expression of proteins related to ferroptosis and autophagy, and ASX pretreatment effectively improved or reversed this condition. According to the biological characteristics of ferroptosis, we confirmed that ASX pretreatment could alleviate ferroptosis and protect the myocardium from DOX-induced toxicity, further validating the results of network pharmacology. Autophagy is a process of cellular degradation and recycling of intracellular components, essential for maintaining cellular homeostasis. It is generally considered a self-regulatory mechanism under normal conditions or various cellular stresses. Many studies have confirmed the role of autophagy in the development of DIC, although its effects can be different or even completely opposite. Some studies have shown that activation of autophagy has a protective effect against DOX-induced cardiotoxicity (Cao et al., 2017; He et al., 2021), whereas others suggest that blocking autophagy can alleviate DIC (He et al., 2022). The differing effects of autophagy on DIC may be related to factors such as the phase of autophagy, DOX dosage and duration, and the dosage, phase, and duration of intervention factors, among others (Galluzzi et al., 2018; Ma W. et al., 2020). In our study, autophagy levels significantly increased in both rat heart tissue and H9C2 cells after DOX exposure, while ASX pretreatment effectively mitigated changes in the expression levels of autophagy-related proteins (P62, Beclin1, LC3). Based on the current findings, we propose that ASX pretreatment can inhibit autophagy and alleviate DOX-induced cardiotoxicity. Interestingly, *in vitro* results showed that the autophagy inhibitor 3-MA displayed a similar ability to inhibit ferroptosis as Fer-1 and ASX, while Fer-1 did not show the same ability to inhibit autophagy as 3-MA and ASX. This suggests that autophagy may play a regulatory role in ferroptosis during the occurrence of DIC (He et al., 2021). ASX may play an anti-DIC role by inhibiting the autophagy process and then affecting the level of ferroptosis. However, this conclusion requires further exploration and experimental validation.

Currently, some novel therapies are being developed to enhance the anti-cancer efficacy of DOX while minimizing its side effects. Nanomedicine delivery is an emerging therapeutic approach (Song et al., 2022; Tu et al., 2023; Yang et al., 2023). DOX-based nanomedicine delivery can significantly mitigate the cardiac toxicity associated with DOX. Song et al. reported that cardiac damage induced by DOX/Au@Pt nanoparticles was significantly alleviated compared to that caused by free DOX (Song et al., 2021). The inhibitory effect of the Au@Pt nanoparticle shell on ROS may be a key factor in alleviating DIC (Yang et al., 2018). Given the close association between ROS and ferroptosis, this suggests that targeting ferroptosis inhibition could represent an important strategy for alleviating DIC. This also inspires us that ASX-based nanomedicine delivery may be a more effective solution to alleviate DIC. It opens up broad research prospects for further enhancing the anti-DIC efficacy of ASX and its potential clinical application value.

In terms of clinical application, the impact of ASX on chemotherapy patients is an important consideration. A previous clinical study demonstrated that a nutritional supplement containing ASX can effectively alleviate chemotherapy-induced toxicity in cancer patients (Ledda et al., 2017). This evidence demonstrates the feasibility of using ASX in clinical settings to alleviate chemotherapy-related side effects. ASX is a safe food supplement, with no severe adverse events reported for the consumption of natural astaxanthin at any dose over any length of time in animals and humans (Davinelli et al., 2019). A toxicological study indicates that ASX doses exceeding 12,000 mg/kg do not produce harmful effects in rats, demonstrating its safety and non-toxicity (Stewart et al., 2008). The potential interactions between ASX and DOX represent an additional consideration during combination therapy. Wakshlag et al. reported that ASX did not affect the killing effect of DOX on canine osteosarcoma cells after combined application (Wakshlag et al., 2010). A study demonstrated that ASX can effectively reduce the toxicity of DOX to normal tissue cells (Krestinin et al., 2024). This suggests that ASX does not interfere with the efficacy of DOX in killing cancer cells, while effectively mitigating DOX-induced damage to normal cells. Additionally, ASX may also potentiate the cytotoxic effects of DOX on cancer cells. Two studies shown that ASX can significantly enhance the cytotoxicity of DOX against breast cancer cells (Fassett and Coombes, 2011; Malhão et al., 2021). Its safety and excellent efficacy demonstrate the great clinical application value of ASX. Furthermore, the properties of ASX must also be considered in clinical applications. ASX possesses strong antioxidant properties but is highly susceptible to oxidation. In the clinical application of ASX to chemotherapy patients, it should be avoided to take oral administration with other oxidizing drugs to prevent the loss of its therapeutic efficacy. Meanwhile, prolonged exposure of ASX to light and air should be avoided. Studies have shown that dissolving ASX in lipids can enhance its stability and bioavailability (Acevedo et al., 2014; Ambati et al., 2014). Light-protected, sealed oil-based capsule are currently the most commonly used methods for administering ASX.

Its favorable safety and efficacy provides considerable flexibility in determining the clinical application dosage of ASX. The recommended or approved safe dosage of ASX in human varies across different countries, ranging from 2 to 24 mg/day (Brendler and Williamson, 2019). Currently, the dosage of ASX varies greatly in the limited clinical studies available, but they generally fall within this range. According to a recent study, oral administration of 4 mg astaxanthin twice daily for 21 consecutive days has been shown to effectively mitigate oxidative damage in cancer patients following cisplatin chemotherapy (Aminullah et al., 2024). The clinical dosing regimen can be adjusted based on the individual circumstances of different chemotherapy patients.

The evidence obtained in this study may provide new insight into the ASX in the prevention and treatment of DIC. Our study also indicated that targeting ferroptosis and autophagy signaling pathways may be an efficient route for alleviating doxorubicin-induced cardiotoxicity. However, there are still several limitations in our research. Firstly, although ASX demonstrates substantial clinical potential in the prevention and treatment of DIC, the current evidence is primarily derived from preclinical studies. Further clinical research is urgently needed to support the translation of ASX into clinical practice. In addition, we only explored the effect of ASX on male rats. The potential gender differences in the effects of ASX remain unknown. Current research indicates that ASX does not exhibit significant gender differences in alleviating oxidative stress, liver damage, and cardiac dysfunction. (Islam et al., 2017; Nakao et al., 2010; Stewart et al., 2008; Wu et al., 2018; Yu et al., 2022; Zhang et al., 2017). However, whether there are gender differences in the effects of ASX on alleviating DIC still requires further experimental investigation. Finally, the animals were pretreated with ASX by oral gavage, which did not fully represent the requirements of ASX-rich food by humans.

## 5 Conclusion

In summary, our research suggests that ASX, a powerful natural phytochemical, can significantly protect cardiomyocytes from DOX-induced oxidative stress and inflammation *in vitro* and *in vivo*. Astaxanthin can mitigate the cardiotoxicity of DOX by attenuating autophagy and ferroptosis. As a potential inhibitor of ferroptosis and autophagy, ASX may offer a viable strategy for preventing and treating DOX-induced heart injury.

## Data Availability

The datasets presented in this study can be found in online repositories. The names of the repository/repositories and accession number(s) can be found in the article/Supplementary Material.

## Abbreviations

DOX, Doxorubicin; ASX, Astaxanthin; DIC, Doxorubicin-induced cardiotoxicity; GEO, Gene Expression Omnibus; DEGs, Differentially expressed genes; KEGG, Kyoto Encyclopedia of Genes and Genomes; GSEA, Gene Set Enrichment Analysis; GSVA, Gene Set Variation Analysis; 3-MA, 3-Methyladenine; Fer-1, Ferrostatin-1; LDH, Lactate dehydrogenase; CK, Creatine kinase; T-AOC, Total antioxidant capacity; GSH, Glutathione; MDA, Malondialdehyde; SOD, Superoxide dismutase; IL-6, Interleukin-6; IL-1β, Interleukin-1β; TNF-α, Tumor Necrosis Factor-α; TFRC, Transferrin receptor 1; FTL, Ferritin light chain; FTH1, Ferritin heavy chain; SLC7A11, Solute carrier family 7 member 11; GPX4, Glutathione peroxidase 4; LC3, Microtubule-associated proteins light chain 3.
