# Supplementary material for: Astaxanthin mitigates doxorubicin-induced cardiotoxicity via inhibiting ferroptosis and autophagy: a study based on bioinformatic analysis and in vivo/vitro experiments
Source: Front Pharmacol. 2025 Jan 21;16:1524448. doi: 10.3389/fphar.2025.1524448 (PMC11790656; doi:10.3389/fphar.2025.1524448)

Fig.7D

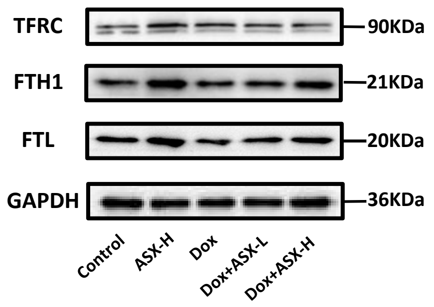

Unedited figures in Fig.7D

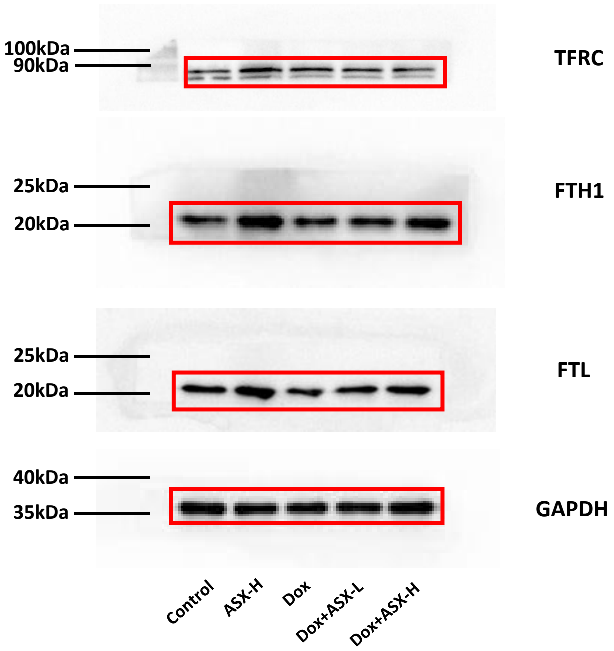

Fig.7H

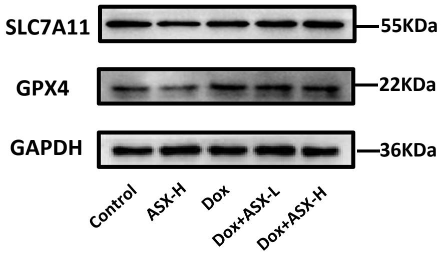

Unedited figures in Fig.7H

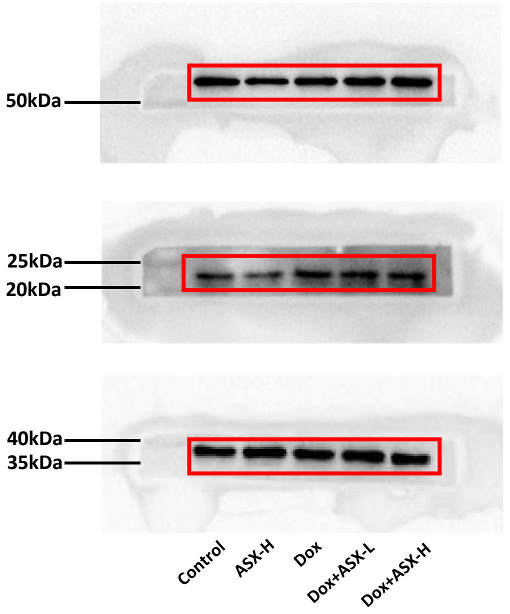

Fig.7K

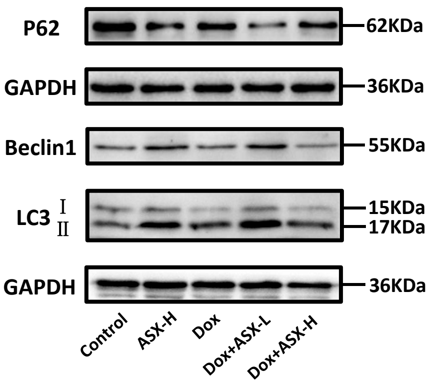

Unedited figures in Fig.7K

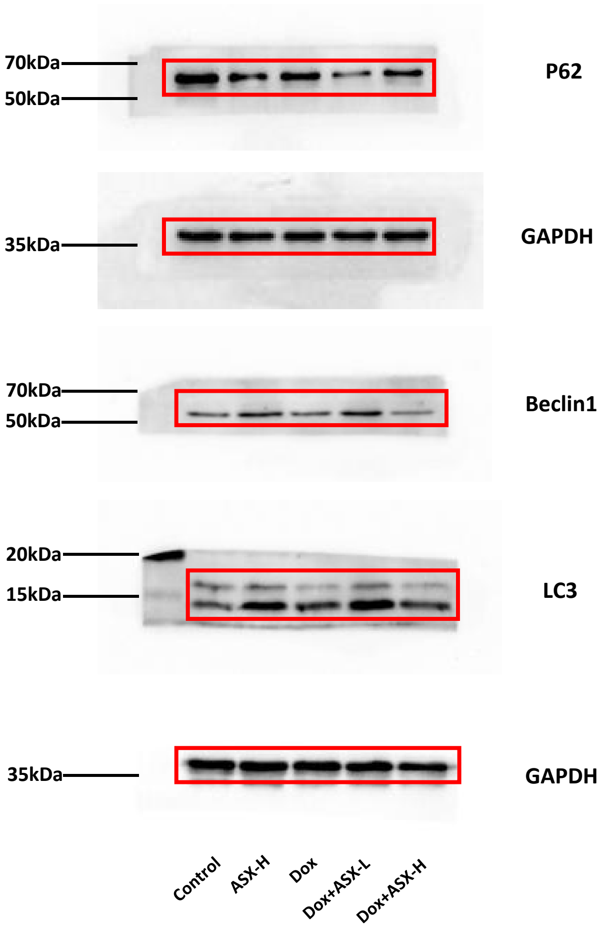

Fig.8D

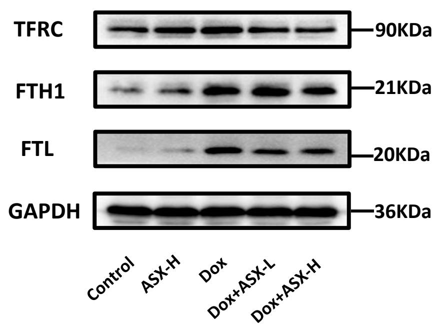

Unedited figures in Fig.8D

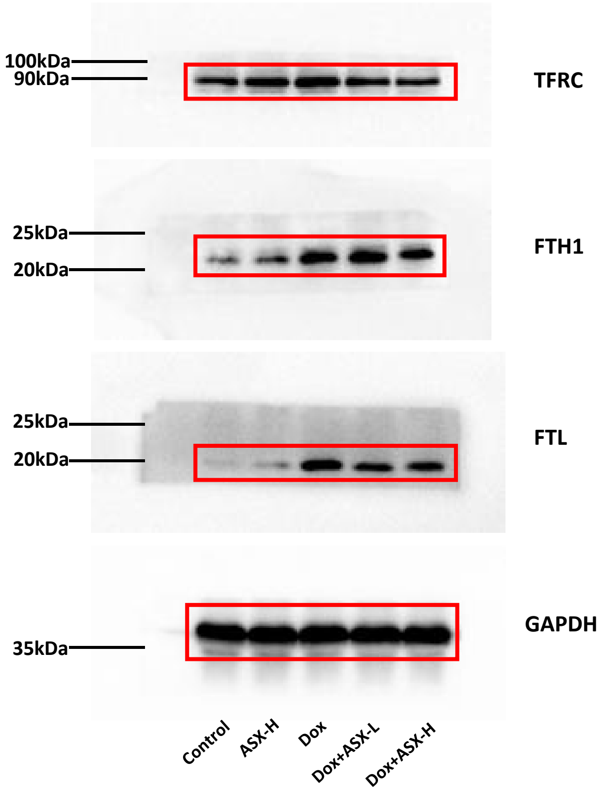

Fig.8H

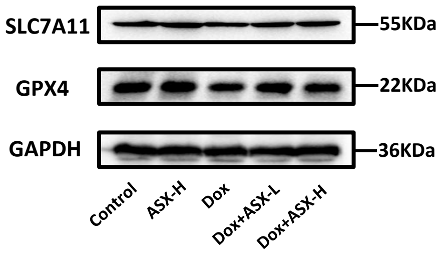

Unedited figures in Fig.8H

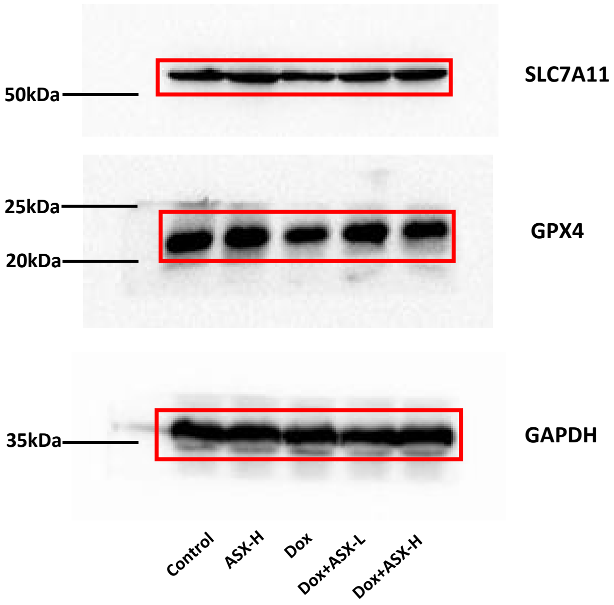

Fig.8K

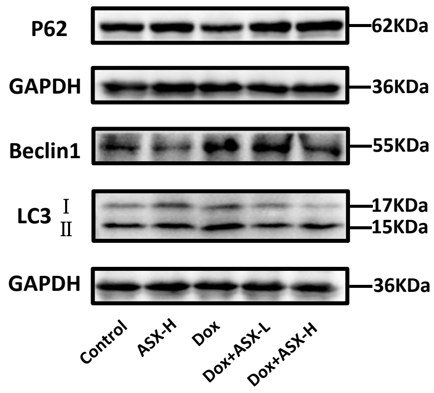

Unedited figures in Fig.8K

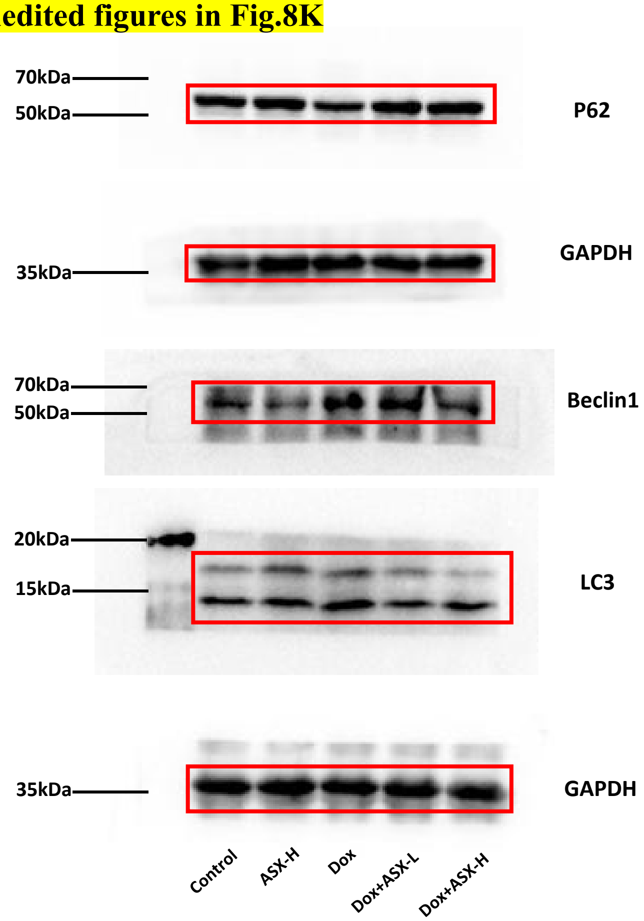

Supplement: Supplementary file 1 [file DataSheet2.pdf]
