# Supplementary material for: Astaxanthin mitigates doxorubicin-induced cardiotoxicity via inhibiting ferroptosis and autophagy: a study based on bioinformatic analysis and in vivo/vitro experiments
Source: Front Pharmacol. 2025 Jan 21;16:1524448. doi: 10.3389/fphar.2025.1524448 (PMC11790656; doi:10.3389/fphar.2025.1524448)
Supplement: Supplementary file 2 [file DataSheet1.docx]

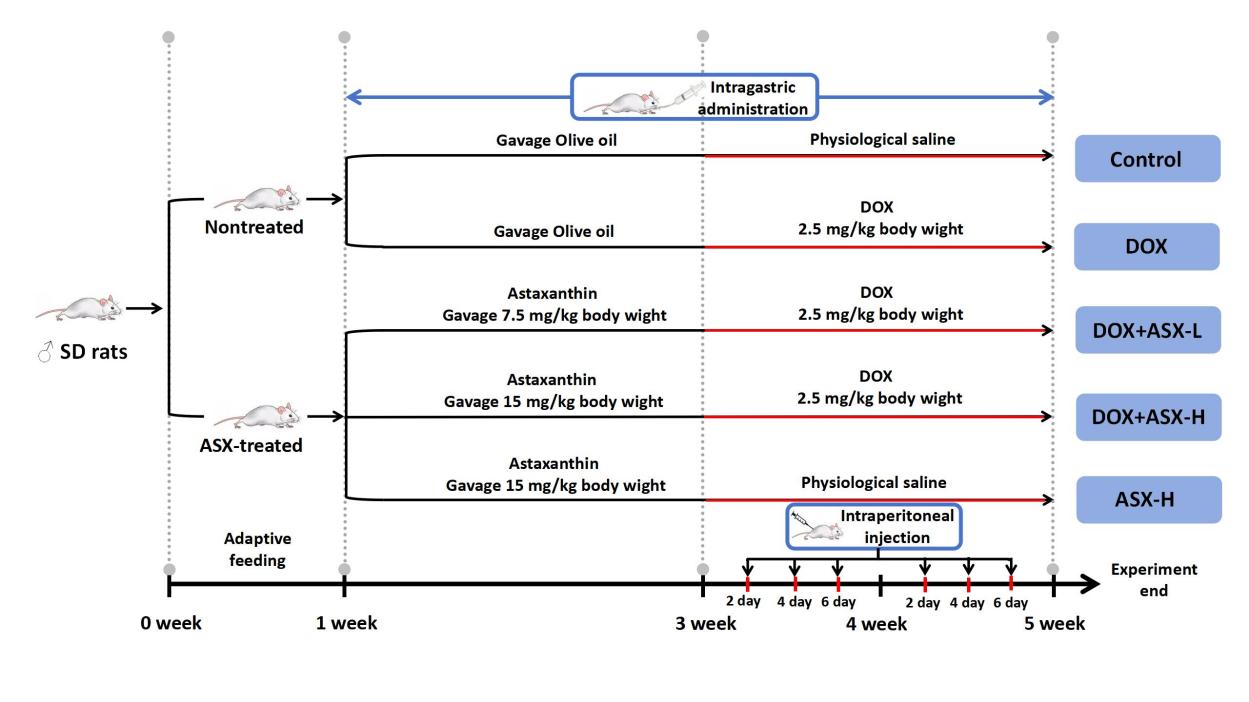


**Figure. S1** Schematic representation of the in vivo experimental design and dosage regimen in this study.

**Table S1** GSEA analysis of DEGs

| **ID** | **Term** | **GeneID** | **Count** | ***p*-value** |
| --- | --- | --- | --- | --- |
| mmu04514 | Cell adhesion molecules - Mus musculus (house mouse) | H2-Q10/Lrrc4c/Cldn10/Slitrk1/H2-Oa/Cdh2/Sdc4/Sele/H2-D1/Itgam/Ptprm/Slitrk3/Ptprs/H2-K1/Cd2/Itga4/Selplg/Pdcd1/Sdc3/Sdc2/Icam2/Itgal/Cntnap1/Cadm1/Jam2/Cd86/Itga9/Vcan/Cd276/Jam3/H2-M3/Negr1/Itga6/Cd99/Itga8/Vcam1/Lrrc4b/Itgb2/Cd274/Ptprc/H2-DMa/Cd34/H2-Eb1/H2-Ab1/H2-Aa | 143 | 1.53342009607761e-07 |
| mmu04145 | Phagosome - Mus musculus (house mouse) | Tlr6/Fcgr2b/Mpo/Cd209c/H2-Q10/Colec12/Atp6v1a/H2-Oa/Cd209e/Thbs3/Msr1/Mrc2/H2-D1/Thbs4/Sec61b/Itgam/Scarb1/Itga2/H2-K1/Mbl1/Atp6v1c2/Comp/Vamp3/Tubb3/Tlr2/Rab5c/Tap1/Ncf2/Colec11/Tubb5/Fcgr1/H2-M3/Cyba/Ncf4/Atp6v0e2/Mrc1/Actg1/Tuba1a/Actb/Itgb2/Cybb/H2-DMa/Ctss/Clec7a/Coro1a/Tuba4a/H2-Eb1/H2-Ab1/H2-Aa/Tfrc | 143 | 1.06117694680816e-06 |
| mmu04140 | Autophagy - animal - Mus musculus (house mouse) | Ddit4/Bnip3/Gabarapl1/Ctsl/Bcl2l1/Sqstm1/Irs2/Pik3r1/Atg13/Wdr45/Pdpk1/Map1lc3b/Tax1bp1/Igf1r/Atg101/Vps16/Mtmr14/Mtmr4/Eif2ak4/Pik3c3/Eif2s1/Mtor/Optn/Ubc/Tsc1/Tbk1/Nbr1/Supt20/Rb1cc1/Wipi2/Bcl2/Vps11/Pten/Deptor/Dapk2/Irs1/Ambra1/Ppp2ca/Vamp8/Atg16l2/Birc6/Igbp1/Lamp2/Pik3ca/Rab7/Tsc2/Rps6kb1/Rraga/Atg9a/Gabarapl2/Dapk3/Dapk1/Ins1 | 156 | 1.0937938027397e-06 |
| mmu04974 | Protein digestion and absorption - Mus musculus (house mouse) | Slc15a1/Slc8a1/Atp1b2/Kcnq1/Fxyd2/Col5a2/Slc36a2/Col8a1/Col1a1/Col1a2/Col14a1/Col5a1/Col6a1/Col6a2/Col15a1/Col6a3/Col3a1 | 96 | 5.69473035801625e-06 |
| mmu04672 | Intestinal immune network for IgA production - Mus musculus (house mouse) | Itga4/Tnfrsf17/Cd86/Cxcl12/Tgfb1/Cxcr4/H2-DMa/Il15/H2-Eb1/H2-Ab1/H2-Aa | 40 | 1.57159697256448e-05 |
| mmu04640 | Hematopoietic cell lineage - Mus musculus (house mouse) | Cd19/Cr2/H2-Oa/Epor/Il3ra/Cd7/Cd3g/Itgam/Il1b/Il9r/Itga2/Cd2/Anpep/Itga4/Cd38/Fcgr1/Ms4a1/Kit/Csf1/Itga6/Gypa/Cd55/Csf1r/H2-DMa/Cd34/Cd24a/H2-Eb1/H2-Ab1/H2-Aa/Tfrc | 85 | 1.36910948721217e-05 |
| mmu04061 | Viral protein interaction with cytokine and cytokine receptor - Mus musculus (house mouse) | Ccr6/Il20/Il20rb/Cxcl10/Il24/Cxcr3/Ackr4/Cx3cr1/Ccl24/Il18/Il10rb/Ackr3/Ccl11/Cxcl5/Cxcl14/Ccr2/Ccl2/Cxcl12/Tnfsf10/Csf1/Il2rg/Ccl7/Cxcr4/Csf1r/Ccl5/Ccr5/Pf4/Ccl12/Ccl8/Cxcl9 | 81 | 1.23328723404157e-05 |
| mmu03320 | PPAR signaling pathway - Mus musculus (house mouse) | Hmgcs2/Plin4/Slc27a1/Angptl4/Cyp27a1/Plin5/Nr1h3/Plin2/Pdpk1/Acox1/Adipoq/Cd36/Apoa1/Pparg/Cpt1a/Ehhadh/Ubc/Acox2/Sorbs1 | 78 | 1.61024279975869e-05 |
| mmu04510 | Focal adhesion - Mus musculus (house mouse) | Lamb1/Itga1/Ptk2/Ilk/Mapk3/Pdgfrb/Arhgap35/Thbs3/Emp2/Fyn/Myl10/Pik3r2/Cav1/Pik3r3/Mapk9/Myl12b/Cav3/Lama3/Thbs4/Grb2/Prkca/Itga2/Lama2/Cav2/Itga4/Kdr/Comp/Vegfa/Myl9/Flna/Vegfc/Parva/Vegfb/Met/Vav3/Rap1b/Mapk10/Akt1/Itga9/Pdgfa/Vtn/Rac2/Lama4/Vasp/Prkcb/Fn1/Itga6/Tnxb/Pdgfc/Actg1/Itga8/Col1a1/Ccnd1/Itgb6/Actb/Igf1/Col1a2/Emp1/Lamb3/Ccnd2/Col6a1/Col6a2/Col6a3/Mylk4 | 194 | 1.53200395445078e-05 |
| mmu04820 | Cytoskeleton in muscle cells - Mus musculus (house mouse) | Thbs3/Lmnb2/Csrp1/Sdc4/Nid2/Myh9/Thbs4/Fmnl2/Sptbn2/Tpm1/Sgcb/Actg2/Itga2/Lama2/Flnc/Itga4/Comp/Fmnl3/Myl9/Tmod1/Sdc3/Sdc2/Plec/Hspg2/Pdlim2/Ampd2/Atp1b2/Dsc2/Myh6/Myl1/Obscn/Tcap/Ank2/Pdlim7/Actc1/Itga9/Eno3/Vcan/Nebl/Ckm/Fn1/Tmod4/Itga6/Fbn1/Sspn/Col5a2/Bgn/Actg1/Itga8/Daam1/Col1a1/Itgb6/Actb/Acta1/Vim/Col1a2/Col5a1/Tnnt1/Tpm4/Fhl2/Col6a1/Col6a2/Lmod3/Col6a3/Col3a1 | 220 | 1.20960362498927e-05 |
| mmu00350 | Tyrosine metabolism - Mus musculus (house mouse) | Comt/Aox1/Fah/Adh1/Got1/Aoc3/Maob | 34 | 6.31200931659805e-05 |
| mmu05416 | Viral myocarditis - Mus musculus (house mouse) | Dmd/H2-Q10/Casp3/H2-Oa/Fyn/Prf1/Cav1/H2-D1/Cav3/Casp8/Sgcb/Lama2/H2-K1/Itgal/Abl1/Myh6/Cd86/Rac2/H2-M3/Sspn/Actg1/Ccnd1/Actb/Cd55/Itgb2/H2-DMa/H2-Eb1/H2-Ab1/H2-Aa | 67 | 6.30839897914967e-05 |
| mmu05152 | Tuberculosis - Mus musculus (house mouse) | Il10/Camk2a/Src/Mapk12/Tgfb2/Cd209b/Mapk14/Bid/Cycs/Nfyc/Ripk2/Cd209a/Fcgr3/Ppp3cb/Nfkb1/Il10ra/Irak1/Itgax/Tlr6/Fcgr2b/Cd209c/Mapk11/Casp3/Mapk3/Camk2g/H2-Oa/Hspd1/Cd209e/Mrc2/Mapk9/Casp8/Itgam/Il18/Il1b/Ifna11/Il10rb/Ciita/Calml4/Tlr2/Rab5c/Mapk10/Akt1/Tgfb3/Fcgr1/Tgfb1/Tlr1/Mrc1/Camp/Fcer1g/Lsp1/Itgb2/H2-DMa/Ctss/Clec7a/Coro1a/Stat1/Ifngr2/Cd74/H2-Eb1/H2-Ab1/H2-Aa | 164 | 6.38189488958636e-05 |
| mmu04062 | Chemokine signaling pathway - Mus musculus (house mouse) | Arrb2/Ccr7/Gnb2/Src/Xcl1/Bcar1/Stat2/Rock2/Arrb1/Adcy3/Cxcr5/Cxcl1/Prex1/Hras/Gng2/Ccr10/Gng4/Adcy2/Gng12/Sos2/Cdc42/Nfkb1/Pik3cg/Kras/Ptk2/Ccr6/Prkaca/Mapk3/Cxcl10/Dock2/Cxcr3/Gng3/Pik3r2/Cx3cr1/Pik3r3/Gnb4/Tiam1/Gnb5/Grb2/Ccl24/Gnb1/Adcy5/Adcy1/Gnai2/Gng7/Cxcl16/Plcg2/Vav3/Rap1b/Ccl11/Cxcl5/Cxcl14/Was/Hck/Ccr2/Akt1/Ccl2/Cxcl12/Rac2/Prkcb/Gng10/Adcy7/Ccl7/Cxcr4/Ccl5/Ccr5/Gng11/Stat1/Pf4/Ccl12/Ccl8/Cxcl9 | 173 | 0.000132140158444249 |
| mmu04015 | Rap1 signaling pathway - Mus musculus (house mouse) | Adcy3/Drd2/Ngfr/Ctnnb1/Hras/Map2k2/Pfn1/Adcy2/Lcp2/Cdc42/Cnr1/Egf/Evl/Sipa1l3/Fgf9/Fgf7/Fyb/Kras/Pard6a/Fgf22/Ralgds/Mapk11/Mapk3/Pdgfrb/F2rl3/Lpar4/Pik3r2/Pik3r3/Tiam1/Itgam/Prkca/Adcy5/Rgs14/Ralb/Tek/Fgf1/Kdr/Vegfa/Calml4/Adcy1/Lpar1/Gnai2/Rapgef5/Itgal/Vegfc/Vegfb/Fgf18/Met/Vav3/Rap1b/Pfn2/Efna1/Akt1/Pdgfa/Sipa1/Rac2/Vasp/Prkcb/Kit/Csf1/Lpar3/Sipa1l2/Pdgfc/Actg1/Rasgrp3/Adcy7/Actb/Igf1/Ngf/Angpt1/Magi3/Itgb2/Map2k6/Csf1r/Fgf16 | 206 | 0.000143541761440898 |
| mmu05322 | Systemic lupus erythematosus - Mus musculus (house mouse) | Cd86/Fcgr1/Trim21/C1qb/C1qa/C1qc/H2-DMa/H2-Eb1/H2-Ab1/H2-Aa | 44 | 0.000189677516987604 |
| mmu05164 | Influenza A - Mus musculus (house mouse) | Casp3/Mapk3/H2-Oa/Cxcl10/Slc25a31/Oas3/Pik3r2/Pik3r3/Tlr3/Casp8/Prkca/Il18/Il1b/Ifna11/Cdk4/Ciita/Bak1/Eif2ak2/Ifih1/Akt1/Ccl2/Irf9/Tnfsf10/Prkcb/Casp1/Pycard/Kpna2/Irf7/Traf3/Oas1a/Actg1/Rsad2/Actb/Ccl5/H2-DMa/Stat1/Ifngr2/H2-Eb1/Ccl12/H2-Ab1/H2-Aa | 153 | 0.000192976979096786 |
| mmu04060 | Cytokine-cytokine receptor interaction - Mus musculus (house mouse) | Ngfr/Cxcr5/Cxcl1/Il9/Tnfrsf4/Tnfsf8/Ccr10/Acvrl1/Acvr1/Ifnar2/Il20ra/Il10ra/Tnfsf4/Il25/Ebi3/Ccr6/Il1rl2/Il20/Il13ra1/Il20rb/Cxcl10/Epor/Tnfrsf19/Il24/Cxcr3/Il3ra/Relt/Ackr4/Cx3cr1/Tnfrsf21/Il1f10/Ccl24/Il18/Prlr/Il1b/Il9r/Ifna11/Il10rb/Il17re/Cxcl17/Ackr3/Tslp/Tnfrsf17/Cxcl16/Ccl11/Cxcl5/Ltb/Cxcl14/Ctf1/Ccr2/Ccl2/Tgfb3/Ghr/Il17d/Bmpr1a/Cxcl12/Tnfsf10/Tgfb1/Csf1/Lepr/Crlf2/Il2rg/Ccl7/Ngf/Cxcr4/Csf1r/Ccl5/Ccr5/Il15/Ifngr2/Pf4/Ccl12/Ccl8/Cxcl9 | 244 | 0.00020812099582469 |
| mmu05140 | Leishmaniasis - Mus musculus (house mouse) | Itgam/Il1b/Itga4/Ptpn6/Tlr2/Ncf2/Marcksl1/Tgfb3/Fcgr1/Tgfb1/Prkcb/Cyba/Ncf4/Itgb2/Cybb/H2-DMa/Stat1/Ifngr2/H2-Eb1/H2-Ab1/H2-Aa | 68 | 0.00024130300373144 |
| mmu03040 | Spliceosome - Mus musculus (house mouse) | Hspa1l/Hspa1a/Srsf5/Sart1/Prpf6/Rbm25/Fus/Ddx5/Thoc2/Usp39/Sf3b1/Tra2a/Syf2/Xab2/Bcas2/Snrnp70/Snrpa1/Thoc1/Srsf7/Snrnp200/Hnrnpu/Hspa1b/Ddx23/Slu7/Snw1/Crnkl1/Sf3b2/Smndc1/Dhx16/Plrg1/Hnrnpa1/Prpf31/Prpf38b/Srsf4/Cdc5l/Dhx15/Srsf10/Cwc15/Ctnnbl1/Prpf19/Acin1/Prpf40a/Ddx42/Prpf40b/Hspa8/Zmat2/Sf3b5/Puf60/Sf3b3/Ddx39b/Cherp | 117 | 0.00026800035733577 |
| mmu00260 | Glycine, serine and threonine metabolism - Mus musculus (house mouse) | Agxt/Pgam2/Gamt/Gatm/Bpgm/Alas1/Alas2 | 37 | 0.0003677280184033 |
| mmu05323 | Rheumatoid arthritis - Mus musculus (house mouse) | Il18/Il1b/Tek/Atp6v1c2/Vegfa/Tlr2/Ctsk/Itgal/Cxcl5/Ltb/Cd86/Ccl2/Tgfb3/Cxcl12/Tgfb1/Csf1/Atp6v0e2/Angpt1/Itgb2/Ccl5/H2-DMa/Il15/H2-Eb1/Ccl12/H2-Ab1/H2-Aa | 83 | 0.000394414105958623 |
| mmu04512 | ECM-receptor interaction - Mus musculus (house mouse) | Thbs3/Sv2a/Sdc4/Lama3/Thbs4/Itga2/Lama2/Itga4/Comp/Hspg2/Itga9/Vtn/Lama4/Fn1/Itga6/Tnxb/Itga8/Col1a1/Itgb6/Col1a2/Frem2/Lamb3/Col6a1/Col6a2/Col6a3 | 86 | 0.000491581270205126 |
| mmu04611 | Platelet activation - Mus musculus (house mouse) | Prkaca/Mapk11/Mapk3/F2rl3/Arhgap35/Fyn/Pik3r2/Pik3r3/Myl12b/P2ry12/Adcy5/Itga2/Adcy1/Gnai2/Orai1/Fermt3/Fgg/Plcg2/Rap1b/Tbxa2r/Btk/Gucy1a2/Akt1/Vasp/Ptgir/Actg1/Fcer1g/Pla2g4a/Adcy7/Col1a1/Actb/Col1a2/Mylk4/Col3a1 | 118 | 0.000492783464146063 |
| mmu00982 | Drug metabolism - cytochrome P450 - Mus musculus (house mouse) | Aox1/Fmo2/Mgst1/Fmo5/Adh1/Gsto2/Gsta2/Gstm1/Fmo1/Gstm2/Gsta3/Maob/Cyp2e1/Gstm4/Mgst3/Gstm6/Gstm5 | 49 | 0.0005655902606255 |
| mmu05145 | Toxoplasmosis - Mus musculus (house mouse) | Mapk10/Akt1/Tgfb3/Tgfb1/Lama4/Alox5/Itga6/Ly96/Ppif/Irgm1/Map2k6/Ccr5/H2-DMa/Lamb3/Stat1/Ifngr2/H2-Eb1/Irgm2/H2-Ab1/H2-Aa/Igtp | 106 | 0.000594403945493363 |
| mmu05332 | Graft-versus-host disease - Mus musculus (house mouse) | H2-T3/H2-Q10/H2-Oa/Prf1/H2-D1/Il1b/H2-K1/Cd86/H2-M3/Gzmb/H2-DMa/H2-Eb1/H2-Ab1/H2-Aa | 33 | 0.000666335659979269 |
| mmu04810 | Regulation of actin cytoskeleton - Mus musculus (house mouse) | Arpc4/Hras/Map2k2/Msn/Pfn1/Actn4/Gng12/Sos2/Cdc42/Egf/Fgf9/Fgf7/Itgax/Kras/C8g/Fgf22/Itga1/Ptk2/Apc2/Mapk3/Pdgfrb/Arhgap35/Lpar4/Brk1/Chrm1/Myl10/Pik3r2/Pik3r3/Myl12b/Tiam1/Myh9/Itgam/Ezr/Itga2/Itga4/Fgf1/Myl9/Lpar1/Arpc5/Itgal/Wasf1/Fgf18/Actr3/Vav3/Pfn2/Akt1/Itga9/Pdgfa/Cxcl12/Tmsb4x/Rac2/Cfl1/Fn1/Itga6/Nckap1l/Pdgfc/Actg1/Arhgef7/Scin/Itga8/Itgb6/Actb/Cxcr4/Itgb2/Fgf16/Cyfip2/Mylk4 | 216 | 0.000658494476093457 |
| mmu05310 | Asthma - Mus musculus (house mouse) | Ccl11/Fcer1g/H2-DMa/H2-Eb1/H2-Ab1/H2-Aa | 23 | 0.000849773523750353 |
| mmu05320 | Autoimmune thyroid disease - Mus musculus (house mouse) | H2-T3/H2-Q10/H2-Oa/Prf1/H2-D1/Ifna11/H2-K1/Cd86/H2-M3/Gzmb/H2-DMa/H2-Eb1/H2-Ab1/H2-Aa | 44 | 0.000945629714002609 |
| mmu04540 | Gap junction - Mus musculus (house mouse) | Gjd2/Adcy3/Drd2/Hras/Map2k2/Adcy2/Sos2/Adrb1/Egf/Itpr1/Kras/Prkaca/Mapk3/Pdgfrb/Grb2/Prkca/Adcy5/Grm1/Tubb3/Adcy1/Lpar1/Gnai2/Tubb5/Gucy1a2/Pdgfa/Prkcb/Pdgfc/Tuba1a/Adcy7/Cdk1/Gja1/Tuba4a | 77 | 0.000956127397832339 |
| mmu04020 | Calcium signaling pathway - Mus musculus (house mouse) | Hrh2/Adcy3/P2rx7/Chrna7/Hrh1/Adrb3/Atp2a2/Ppp3cb/Adcy2/Gna14/Hrc/Adrb1/Egf/Vdac3/Fgf9/Fgf7/Itpr1/Fgf22/Camk1g/Prkaca/Cysltr1/Tfeb/Camk2g/Pdgfrb/Erbb3/Slc25a31/Chrm1/Avpr1b/Tpcn2/Ptgfr/Pde1c/Prkca/Grm1/Fgf1/Kdr/Vegfa/Calml4/Adcy1/Cd38/Orai1/Casq1/Slc8a1/Ryr2/Vegfc/Cacna1h/Vegfb/Fgf18/Adra1b/Met/Plcg2/Camk1/Tbxa2r/Agtr1a/Itpkb/Asph/Pdgfa/Ret/Prkcb/Pdgfc/Ppif/Adcy7/Ngf/Casq2/Cxcr4/Cacna1g/Fgf16/Mylk4 | 239 | 0.00103314476330448 |
| mmu04014 | Ras signaling pathway - Mus musculus (house mouse) | Hras/Gng2/Gng4/Map2k2/Gng12/Pla1a/Sos2/Cdc42/Egf/Ksr2/Nfkb1/Fgf9/Fgf7/Kras/Fgf22/Rgl1/Ralgds/Prkaca/Arf6/Mapk3/Pdgfrb/Gng3/Pik3r2/Pik3r3/Mapk9/Gnb4/Tiam1/Gnb5/Igf2/Grb2/Prkca/Gnb1/Ralb/Tek/Fgf1/Kdr/Rasgrf2/Vegfa/Calml4/Rab5c/Rapgef5/Gng7/Vegfc/Vegfb/Fgf18/Met/Plcg2/Abl1/Rap1b/Ntf3/Efna1/Mapk10/Akt1/Pdgfa/Ets1/Rac2/Prkcb/Kit/Csf1/Ralgapa2/Pla2g2d/Gng10/Pdgfc/Rasgrp3/Pla2g4a/Igf1/Ngf/Angpt1/Csf1r/Gng11/Fgf16/Pla2g5 | 221 | 0.0011147938264984 |
| mmu05150 | Staphylococcus aureus infection - Mus musculus (house mouse) | Mbl1/Selplg/Itgal/Fgg/Fcgr1/Camp/C1qb/C1qa/Itgb2/C1qc/H2-DMa/C3ar1/H2-Eb1/H2-Ab1/H2-Aa | 84 | 0.00123139468896171 |
| mmu00430 | Taurine and hypotaurine metabolism - Mus musculus (house mouse) | Cdo1/Fmo2/Fmo5/Fmo1/Csad | 17 | 0.00139471267517542 |
| mmu03008 | Ribosome biogenesis in eukaryotes - Mus musculus (house mouse) | Rasl2-9/Wdr75/Gnl2/Rpp40/Sbds/Gtpbp4/Mphosph10/Nop58/Lsg1/Nxf1/Wdr43/Heatr1/Drosha/Imp3/Nob1/Xrn1/Rpp25/Nxf7/Riok2/Gnl3l/Tbl3/Nvl/Riok1/Gar1/Imp4/Rbm28/Rrp7a/Eif6/Gnl3/Wdr36/Wdr3/Pwp2/Nmd3/Xrn2 | 70 | 0.00143309842829898 |
| mmu01524 | Platinum drug resistance - Mus musculus (house mouse) | Cdkn1a/Bcl2l1/Mgst1/Fas/Pik3r1/Pdpk1/Xpa/Gsto2/Gsta2/Msh2/Atp7a/Gstm1/Gstm2/Birc2/Birc3/Map3k5/Ercc1/Bcl2/Erbb2/Gsta3/Gstm4/Pik3ca/Mgst3/Gstm6 | 75 | 0.00143013748113307 |
| mmu04940 | Type I diabetes mellitus - Mus musculus (house mouse) | H2-T3/H2-Q10/H2-Oa/Hspd1/Prf1/H2-D1/Il1b/H2-K1/Cd86/H2-M3/Gzmb/H2-DMa/H2-Eb1/H2-Ab1/H2-Aa | 42 | 0.00158537835321649 |
| mmu01240 | Biosynthesis of cofactors - Mus musculus (house mouse) | Gmppb/Kmo/Nme7/Ugt2b1/Alad/Fpgs/Aspdh/Haao/Qprt/Nme4/Fech/Ak8/Ugdh/Nmnat2/Shmt1/Nme6/Coq7/Coq5/Pmm2/Ggcx/Ak1/Ggh/Mthfd2l/Ido1/Nme1/Cad/Rgn/Pnpo/Cox10/Ido2/Mocs2/Dhodh/Mpi/Vkorc1/Ppcs/Cmpk2/Aldh1b1/Nmnat3/Pdxk/Alpl/Lipt2/Mthfd1l/Ak4/Gusb/Afmid/Alas1/Alas2 | 132 | 0.00157547449328525 |
| mmu05330 | Allograft rejection - Mus musculus (house mouse) | H2-T3/H2-Q10/H2-Oa/Prf1/H2-D1/H2-K1/Cd86/H2-M3/Gzmb/H2-DMa/H2-Eb1/H2-Ab1/H2-Aa | 35 | 0.0016907474683228 |
| mmu04260 | Cardiac muscle contraction - Mus musculus (house mouse) | Cacna1f/Slc9a1/Uqcr10/Cox7a2l/Atp1b1/Cox4i2/Cacna2d2/Tnni3/Uqcrfs1/Cox8a/Cacnb4/Atp2a2/Cacna2d1/Hrc/Atp1b3/Tpm2/Cox8c/Tpm1/Cox6a1/Cox7a1/Cyc1/Slc8a1/Ryr2/Slc9a7/Atp1b2/Uqcrc1/Cacng8/Myh6/Actc1/Asph/Fxyd2/Casq2/Tpm4/Cacnb2 | 82 | 0.00207699832086814 |
| mmu04612 | Antigen processing and presentation - Mus musculus (house mouse) | H2-K1/Ciita/Tap1/B2m/Lgmn/Ctsb/H2-M3/Ifi30/Psme1/H2-DMa/Ctss/Cd74/H2-Eb1/H2-Ab1/H2-Aa | 57 | 0.00223624699717471 |
| mmu05144 | Malaria - Mus musculus (house mouse) | Thbs3/Sele/Thbs4/Il18/Il1b/Comp/Tlr2/Sdc2/Itgal/Met/Ccl2/Tgfb3/Tgfb1/Gypa/Vcam1/Itgb2/Ccl12/Hbb-b2 | 49 | 0.00260376277839196 |
| mmu05414 | Dilated cardiomyopathy - Mus musculus (house mouse) | Tgfb2/Sgce/Cacna2d2/Mybpc3/Tnni3/Adcy3/Cacnb4/Atp2a2/Cacna2d1/Adcy2/Adrb1/Dmd/Itga1/Prkaca/Tpm2/Cav3/Tpm1/Sgcb/Adcy5/Itga2/Lama2/Itga4/Adcy1/Slc8a1/Ryr2/Cacng8/Myh6/Actc1/Itga9/Tgfb3/Tgfb1/Itga6/Sspn/Actg1/Adcy7/Itga8/Itgb6/Actb/Igf1/Tpm4/Cacnb2 | 101 | 0.00273052008966702 |
| mmu05202 | Transcriptional misregulation in cancer - Mus musculus (house mouse) | Cdkn1a/Mmp3/Zbtb16/Bcl2l1/Prom1/Mllt3/Cebpb/Il1r2/H3f3b/Igfbp3/Kmt2a/Flt1/Myc/Etv5/Ddb2/Igf1r/H3f3a/Wt1/Nfkbiz/Fus/Pparg/Ddx5/Slc45a3/Bcl6/Id2/Tgfbr2/Birc2/Gadd45g/Mmp9/Gadd45b/Birc3/Taf15/Aspscr1/Ccnt2/Kdm6a/Il3/Klf3/Ncor1/Sp1/Cdkn1b | 166 | 0.00278899853128184 |
| mmu05410 | Hypertrophic cardiomyopathy - Mus musculus (house mouse) | Cav3/Tpm1/Sgcb/Itga2/Lama2/Prkag2/Itga4/Slc8a1/Ryr2/Cacng8/Myh6/Actc1/Itga9/Tgfb3/Tgfb1/Itga6/Prkab1/Sspn/Actg1/Itga8/Itgb6/Actb/Igf1/Tpm4/Cacnb2 | 97 | 0.00286050940109354 |
| mmu04966 | Collecting duct acid secretion - Mus musculus (house mouse) | Atp6v1a/Atp4b/Atp6v1c2/Slc12a7/Car2/Atp6v0e2/Slc4a1 | 26 | 0.00379018299212663 |
| mmu04933 | AGE-RAGE signaling pathway in diabetic complications - Mus musculus (house mouse) | Pik3r2/Egr1/Sele/Pik3r3/Mapk9/Prkca/Stat5a/Il1b/Cdk4/Vegfa/Vegfc/Smad3/Vegfb/Plcg2/Agtr1a/Mapk10/Akt1/Ccl2/Tgfb3/Tgfb1/Prkcb/Fn1/Col1a1/Ccnd1/Vcam1/Col1a2/Cybb/Stat1/Ccl12/Col3a1 | 98 | 0.00408740121343388 |
| mmu04371 | Apelin signaling pathway - Mus musculus (house mouse) | Hras/Gng2/Gng4/Map2k2/Adcy2/Gng12/Pik3r4/Pik3cg/Itpr1/Notch3/Kras/Prkaca/Prkce/Mapk3/Mef2a/Gng3/Egr1/Gnb4/Gnb5/Tfam/Gnb1/Adcy5/Prkag2/Hdac4/Calml4/Adcy1/Gnai2/Slc8a1/Ryr2/Gng7/Smad3/Mef2c/Agtr1a/Acta2/Akt1/Plat/Prkab1/Gng10/Adcy7/Ccnd1/Pde3b/Apln/Gng11/Mylk4/Aplnr | 128 | 0.00411902520734826 |
| mmu04971 | Gastric acid secretion - Mus musculus (house mouse) | Atp1a2/Chrm3/Adcy8/Kcnk10/Camk2b/Slc9a1/Camk2a/Atp1b1/Gast/Hrh2/Adcy3/Adcy2/Atp1b3/Itpr1/Prkaca/Camk2g/Atp4b/Prkca/Adcy5/Ezr/Calml4/Adcy1/Gnai2/Atp1b2/Prkcb/Kcnq1/Car2/Actg1/Kcnk2/Adcy7/Actb/Mylk4 | 71 | 0.00560818541423779 |
| mmu04666 | Fc gamma R-mediated phagocytosis - Mus musculus (house mouse) | Arf6/Prkce/Mapk3/Pik3r2/Pik3r3/Prkca/Arpc5/Wasf1/Actr3/Plcg2/Vav3/Marcksl1/Was/Hck/Akt1/Fcgr1/Inppl1/Rac2/Vasp/Prkcb/Cfl1/Marcks/Pla2g4a/Asap3/Scin/Ptprc | 87 | 0.00584954796759338 |
| mmu05165 | Human papillomavirus infection - Mus musculus (house mouse) | Hdac1/Map2k2/Wnt2/Sos2/Cdc42/Ifnar2/E2f1/Egf/Nfkb1/Col4a4/H2-T3/Llgl1/Hes2/Fzd7/Notch3/Rbl1/Ppp2r1a/Kras/Pard6a/Lamb1/Itga1/Ptk2/H2-Q10/Prkaca/Apc2/Atp6v1a/Casp3/Rfng/Wnt8a/Mapk3/Pdgfrb/Thbs3/Mfng/Pik3r2/Pik3r3/H2-D1/Lama3/Tlr3/Thbs4/Casp8/Grb2/Dvl3/Ifna11/Itga2/Lama2/H2-K1/Hes7/Ptger4/Cdk4/Itga4/Atp6v1c2/Wnt10b/Comp/Vegfa/Fzd2/Trp53/Wnt5b/Irf1/Wnt7a/Bak1/Eif2ak2/Hes6/Ccna2/Ppp2r3a/Pkm/Akt1/Itga9/Irf9/Heyl/Wnt5a/Vtn/Lama4/H2-M3/Fn1/Oasl2/Itga6/Traf3/Tnxb/Atp6v0e2/Itga8/Col1a1/Ccnd1/Itgb6/Col1a2/Lamb3/Ccnd2/Stat1/Col6a1/Col6a2/Col6a3 | 317 | 0.00655018903984079 |
| mmu04151 | PI3K-Akt signaling pathway - Mus musculus (house mouse) | Mapk3/Pdgfrb/Rptor/Erbb3/Phlpp1/Epor/Lpar4/Thbs3/Chrm1/Il3ra/Tcl1/Gng3/G6pc2/Pik3r2/Pik3r3/Gnb4/Lama3/Gnb5/Igf2/Thbs4/Grb2/Prkca/Gnb1/Prlr/Ywhaq/Ifna11/Itga2/Lama2/Lpar6/Tek/Cdk4/Itga4/Fgf1/Kdr/Comp/Vegfa/Lpar1/Tlr2/Trp53/Gng7/Vegfc/Vegfb/Fgf18/Met/G6pc3/Ppp2r3a/Nrtn/Ntf3/Efna1/Akt1/Itga9/Ghr/Pdgfa/Ret/Vtn/Eif4e/Lama4/Kit/Csf1/Fn1/Itga6/Lpar3/Tnxb/Gng10/Pdgfc/Itga8/Il2rg/Col1a1/Ccnd1/Itgb6/Igf1/Ngf/Pik3ap1/Angpt1/Col1a2/Csf1r/Gng11/Lamb3/Ccnd2/Col6a1/Col6a2/Fgf16/Col6a3 | 342 | 0.00646367710912592 |
| mmu04137 | Mitophagy - animal - Mus musculus (house mouse) | Bnip3/Gabarapl1/Bcl2l1/Sqstm1/Foxo3/Map1lc3b/Tbc1d15/Tax1bp1/Tbc1d17/Cited2/Eif2s1/Optn/Ubc/Tbk1/Siah1a/Usp15/Pink1/Nbr1/Jun/Sp1/Ambra1/Tomm7/Rab7/Atf4/Atg9a/Gabarapl2/Mul1 | 92 | 0.00736884112694356 |
| mmu04650 | Natural killer cell mediated cytotoxicity - Mus musculus (house mouse) | Hras/Map2k2/Ppp3cb/Sos2/Lcp2/Ifnar2/Kras/Casp3/Mapk3/Fyn/Pik3r2/Prf1/Pik3r3/Sh3bp2/H2-D1/Grb2/Prkca/Ifna11/H2-K1/Ptpn6/Icam2/Itgal/Plcg2/Vav3/Tnfsf10/Rac2/Prkcb/Fcer1g/Itgb2/Gzmb/Tyrobp/Ifngr2/Cd48 | 99 | 0.00740057206942737 |
| mmu05215 | Prostate cancer - Mus musculus (house mouse) | Cdkn1a/Mmp3/Il1r2/Pik3r1/Nfkbia/Pdpk1/Etv5/Igf1r/Hsp90aa1/Mtor/Mmp9/Chuk/Braf/Rb1/Bcl2/Pdgfd/Erbb2/Hsp90b1/Cdkn1b/Pten/Foxo1/Crebbp/Pik3ca/Ikbkb/Atf4/Araf/Insrr | 97 | 0.00733714549242241 |
| mmu04068 | FoxO signaling pathway - Mus musculus (house mouse) | Cdkn1a/Bnip3/Gabarapl1/Irs2/Fbxo32/Pik3r1/Rbl2/Sgk1/Foxo3/Pdpk1/Cat/S1pr1/Igf1r/Stat3/Ccng2/Insr/Setd7/Bcl6/Tgfbr2/Gadd45g/Plk2/Chuk/Gadd45b/Braf/Klf2/Sirt1/Ccnb1/Cdkn1b/Agap2/Pten/Foxo1/Irs1/Pck2/Cdkn2d/Crebbp/Pik3ca/Ikbkb/Araf/Foxo4 | 129 | 0.00713976006592424 |
| mmu04670 | Leukocyte transendothelial migration - Mus musculus (house mouse) | Myl10/Pik3r2/Pik3r3/Myl12b/Itgam/Prkca/Ezr/Ctnna1/Itga4/Myl9/Gnai2/Itgal/Plcg2/Ncf2/Vav3/Jam2/Rap1b/Thy1/Cxcl12/Sipa1/Rac2/Jam3/Vasp/Prkcb/Cyba/Ncf4/Actg1/Cd99/Actb/Vcam1/Cxcr4/Itgb2/Cybb | 111 | 0.00768333328690875 |
| mmu05321 | Inflammatory bowel disease - Mus musculus (house mouse) | Il18/Il1b/Tlr2/Smad3/Tgfb3/Tgfb1/Il2rg/H2-DMa/Stat1/Ifngr2/H2-Eb1/H2-Ab1/H2-Aa | 55 | 0.00819689405346063 |
| mmu04621 | NOD-like receptor signaling pathway - Mus musculus (house mouse) | Mapk12/Xiap/Mfn2/Atg5/Stat2/Mapk14/Panx1/Gsdmd/Trpv2/P2rx7/Defa-rs10/Cxcl1/Gbp2b/Ripk2/Dnm1l/Sharpin/Ifnar2/Naip5/Nfkb1/Vdac3/Txn2/Pkn1/Itpr1/Nod1/Mapk11/Aim2/Mapk3/Ripk3/Oas3/Mapk9/Casp8/Tnfaip3/Il18/Il1b/Ifna11/Antxr1/Ctsb/Trip6/Mapk10/Ccl2/Irf9/Rbck1/Gpsm3/Casp1/Cyba/Antxr2/Pycard/Irf7/Traf3/Oas1a/Gbp7/Camp/Irgm1/Ccl5/Cybb/Gbp2/Stat1/Gbp3/Ccl12/Irgm2 | 164 | 0.00903354934681614 |
| mmu00190 | Oxidative phosphorylation - Mus musculus (house mouse) | Ndufa13/Ndufa9/Ndufb8/Ndufs7/Ndufs5/Atp6ap1/Atp6v0e/Ndufb7/Uqcrq/Uqcr10/Cox7a2l/Ndufa8/Atp12a/Cox4i2/Uqcrfs1/Cox8a/Cycs/Ppa2/Sdhb/Ndufv3/Sdhd/Atp6v1a/Cox8c/Atp4b/Cox11/Ndufc2/Ndufv2/Ndufs4/Cox6a1/Cox10/Atp6v1c2/Ndufab1/Cox7a1/Ndufa4l2/Ndufb10/Cyc1/Lhpp/Ppa1/Uqcrc1/Ndufs8/Atp6v0e2/Ndufb2 | 102 | 0.00925157988430601 |
| mmu05415 | Diabetic cardiomyopathy - Mus musculus (house mouse) | Sdhb/Mapk11/Ndufv3/Sdhd/Mpc2/Camk2g/Cox8c/Slc25a31/Ndufc2/Pik3r2/Pik3r3/Mapk9/Ndufv2/Ndufs4/Prkca/Cox6a1/Ndufab1/Cox7a1/Ndufa4l2/Ndufb10/Cyc1/Ryr2/Smad3/Pdhb/Ncf2/Uqcrc1/Agtr1a/Mapk10/Slc2a4/Akt1/Tgfb3/Ndufs8/Tgfb1/Rac2/Prkcb/Cyba/Ncf4/Ppif/Col1a1/Ndufb2/Col1a2/Cybb/Col3a1 | 172 | 0.0108492912252164 |
| mmu05133 | Pertussis - Mus musculus (house mouse) | Nfkb1/Irak1/Nod1/Mapk11/Casp3/Mapk3/Zp3r/Mapk9/Itgam/Il1b/Calml4/Gnai2/Irf1/Casp7/Irf8/Cxcl5/Mapk10/Casp1/Cfl1/Pycard/Ly96/C1qb/C1qa/Itgb2/C1qc | 67 | 0.0133584773414898 |
| mmu04931 | Insulin resistance - Mus musculus (house mouse) | Socs3/Slc27a1/Irs2/Nr1h3/Pik3r1/Ptprf/Nfkbia/Pdpk1/Ptpn11/Stat3/Pygl/Tbc1d4/Acacb/Cd36/Insr/Cpt1a/Mtor/Agt/Mlxip/Slc2a1/Trib3/Gfpt2/Pten/Foxo1/Irs1/Pck2/Pik3ca/Ikbkb/Ppp1cb/Prkcz/Rps6kb1 | 104 | 0.0141012341916933 |
| mmu04261 | Adrenergic signaling in cardiomyocytes - Mus musculus (house mouse) | Cacna1f/Slc9a1/Adrb2/Atf6b/Camk2a/Atp1b1/Mapk12/Creb3l2/Ppp2r1b/Cacna2d2/Tnni3/Mapk14/Adcy3/Cacnb4/Atp2a2/Cacna2d1/Adcy2/Adrb1/Atp1b3/Pik3cg/Ppp2r1a/Prkaca/Mapk11/Tpm2/Mapk3/Camk2g/Bves/Tpm1/Prkca/Adcy5/Calml4/Adcy1/Gnai2/Slc8a1/Ryr2/Adra1b/Ppp2r3a/Atp1b2/Agtr1a/Cacng8/Myh6/Akt1/Actc1/Kcne1/Scn4b/Kcnq1/Fxyd2/Scn5a/Kcnk2/Adcy7/Popdc2/Tpm4/Cacnb2 | 148 | 0.0139567460476928 |
| mmu05204 | Chemical carcinogenesis - DNA adducts - Mus musculus (house mouse) | Sult1a1/Cyp1b1/Mgst1/Ephx1/Ptgs2/Gsto2/Gsta2/Hsd11b1/Gstm1/Gstm2/Gsta3/Cyp2e1/Gstm4/Mgst3/Gstm6/Nat3/Gstm5 | 57 | 0.0161282494993384 |
| mmu04010 | MAPK signaling pathway - Mus musculus (house mouse) | Map2k2/Cacna2d1/Ppp3cb/Gng12/Sos2/Cdc42/Egf/Nfkb1/Fgf9/Irak1/Fgf7/Ecsit/Map4k1/Kras/Fgf22/Dusp2/Mapk8ip1/Prkaca/Mapk11/Dusp3/Casp3/Mapk3/Pdgfrb/Erbb3/Mapk9/Igf2/Grb2/Srf/Prkca/Il1b/Mapkapk5/Tek/Ptprr/Fgf1/Kdr/Rasgrf2/Vegfa/Rps6ka1/Trp53/Relb/Rps6ka2/Flna/Vegfc/Cacna1h/Vegfb/Fgf18/Met/Mef2c/Ddit3/Rap1b/Nrtn/Ntf3/Cacng8/Efna1/Mapk10/Akt1/Tgfb3/Pdgfa/Ret/Dusp7/Tgfb1/Rac2/Prkcb/Kit/Csf1/Dusp6/Dusp10/Pdgfc/Rasgrp3/Pla2g4a/Gadd45a/Igf1/Ngf/Mapkapk3/Angpt1/Cacna1g/Map2k6/Csf1r/Cacnb2/Fgf16 | 290 | 0.0161622847286326 |
| mmu05340 | Primary immunodeficiency - Mus musculus (house mouse) | Cd79a/Ciita/Orai1/Tap1/Btk/Il2rg/Blnk/Ptprc/Ung | 36 | 0.0166068269412079 |
| mmu04714 | Thermogenesis - Mus musculus (house mouse) | Ndufb8/Ndufs7/Adcy8/Ndufs5/Ndufb7/Nras/Uqcrq/Sirt6/Arid1b/Smarcd1/Fgf21/Cox20/Uqcr10/Prkab2/Cox7a2l/Ndufa8/Prkaa2/Mapk12/Cox4i2/Creb3l2/Mapk14/Adcy3/Uqcrfs1/Cox8a/Hras/Adrb3/Ndufaf3/Adcy2/Sos2/Cnr1/Dpf3/Kras/Sdhb/Prkaca/Mapk11/Ndufv3/Sdhd/Actl6b/Ndufaf4/Rptor/Cox8c/Cox11/Ndufc2/Slc25a29/Ndufaf5/Ndufv2/Mgll/Grb2/Ndufs4/Coa6/Adcy5/Cox6a1/Coa3/Cox10/Prkag2/Ndufab1/Cox7a1/Ndufa4l2/Ndufaf1/Adcy1/Rps6ka1/Ndufb10/Cyc1/Rps6ka2/Smarcd3/Uqcrc1/Coa5/Ndufaf6/Acsl5/Ndufs8/Nppb/Prkab1/Actg1/Adcy7/Ndufb2/Actb | 194 | 0.0176262417795815 |
| mmu04613 | Neutrophil extracellular trap formation - Mus musculus (house mouse) | Mapk14/Hdac9/Gsdmd/Fcgr3/Hdac1/Map2k2/Nfkb1/Vdac3/Clcn3/Mpo/Elane/Mapk11/Aqp9/Mapk3/Slc25a31/Pik3r2/Pik3r3/Padi4/Itgam/Prkca/Hdac4/Selplg/Tlr2/Itgal/Fgg/Plcg2/Ncf2/Akt1/Fcgr1/Rac2/Prkcb/Casp1/Cyba/Ncf4/Ppif/Camp/Actg1/Actb/Itgb2/Cybb/Clec7a | 106 | 0.0183399766847665 |
| mmu04658 | Th1 and Th2 cell differentiation - Mus musculus (house mouse) | Jak3/Nfatc2/Rbpjl/Notch2/Il12a/H2-Ob/Zap70/Mapk12/Mapk14/Rbpj/Cd3d/Ppp3cb/Nfkb1/Nfkbie/Notch3/Mapk11/Mapk3/Dll1/H2-Oa/Mapk9/Dll4/Cd3g/Stat5a/Mapk10/Il2rg/H2-DMa/Stat1/Ifngr2/H2-Eb1/H2-Ab1/H2-Aa | 85 | 0.0185187288941697 |
| mmu04926 | Relaxin signaling pathway - Mus musculus (house mouse) | Atf6b/Arrb2/Gnb2/Src/Mapk12/Creb3l2/Mapk14/Arrb1/Adcy3/Hras/Gng2/Gng4/Map2k2/Adcy2/Gng12/Sos2/Nfkb1/Col4a4/Kras/Prkaca/Mapk11/Mapk3/Gng3/Pik3r2/Pik3r3/Mapk9/Gnb4/Gnb5/Grb2/Prkca/Mmp13/Gnb1/Adcy5/Vegfa/Adcy1/Gnai2/Gng7/Vegfc/Vegfb/Acta2/Mapk10/Akt1/Tgfb1/Gng10/Adcy7/Col1a1/Col1a2/Gng11/Col3a1 | 122 | 0.0186276625935472 |
| mmu04340 | Hedgehog signaling pathway - Mus musculus (house mouse) | Arrb2/Smurf1/Dhh/Smo/Arrb1/Gli3/Csnk1g2/Gli1/Prkaca/Ptch2/Cdon/Gas1/Hhatl/Ccnd1/Scube2/Ccnd2 | 50 | 0.0192994257076359 |
| mmu03260 | Virion - Human immunodeficiency virus - Mus musculus (house mouse) | Cd209b/Cd209a/Cd209c/Cd209e/Cxcr4/Ccr5 | 10 | 0.020699530179095 |
| mmu04662 | B cell receptor signaling pathway - Mus musculus (house mouse) | Hras/Map2k2/Ppp3cb/Sos2/Cd81/Nfkb1/Fcgr2b/Nfkbie/Kras/Cd19/Cr2/Mapk3/Pik3r2/Pik3r3/Grb2/Cd79a/Ptpn6/Cd79b/Plcg2/Vav3/Blk/Btk/Akt1/Cd72/Inppl1/Rac2/Prkcb/Rasgrp3/Pik3ap1/Blnk/Bank1 | 71 | 0.021799952628798 |
| mmu05412 | Arrhythmogenic right ventricular cardiomyopathy - Mus musculus (house mouse) | Dmd/Itga1/Dsg2/Cdh2/Cav3/Sgcb/Itga2/Lama2/Ctnna1/Itga4/Slc8a1/Ryr2/Cacng8/Dsc2/Itga9/Itga6/Sspn/Actg1/Itga8/Itgb6/Actb/Gja1/Cacnb2 | 84 | 0.0221477838360755 |
| mmu00860 | Porphyrin metabolism - Mus musculus (house mouse) | Cox10/Blvra/Blvrb/Hccs/Gusb/Alas1/Alas2 | 33 | 0.0244945362154423 |
| mmu05020 | Prion disease - Mus musculus (house mouse) | Psmd14/C8g/Psmb3/Sdhb/Prkaca/Mapk11/Ndufv3/Sdhd/Casp3/Mapk3/Cox8c/Slc25a31/Psmb2/Ndufc2/Fyn/Pik3r2/Cav1/Egr1/Pik3r3/Mapk9/Cav3/Ndufv2/Psmb7/Ndufs4/Psmb6/Il1b/Cox6a1/Cav2/Ndufab1/Cox7a1/Stip1/Ndufa4l2/Tubb3/Ndufb10/Cyc1/Ryr2/Ncf2/Ddit3/Uqcrc1/Tubb5/Mapk10/Ndufs8/Rac2/Cyba/Ncf4/Ppif/Tuba1a/C1qb/Ndufb2/C1qa/Ccl5/Cybb/C1qc/Tuba4a | 232 | 0.0246429221040168 |
| mmu05166 | Human T-cell leukemia virus 1 infection - Mus musculus (house mouse) | Cd40/Xiap/Lta/Tgfb2/Creb3l2/Chek1/Il15ra/Tspo/Calr/Tcf3/H2-T24/Adcy3/Msx1/Hras/Cd3d/Cdc20/Map2k2/Ppp3cb/Msx2/Adcy2/E2f1/Anapc10/Nfkb1/Vdac3/Bub1b/H2-T3/Kras/Cdkn2c/H2-Q10/Prkaca/Cdkn2b/Mapk3/H2-Oa/Slc25a31/Pik3r2/Egr1/Pik3r3/Mapk9/H2-D1/Srf/Cd3g/Stat5a/Adcy5/H2-K1/Cdk4/Adcy1/E2f2/Trp53/Relb/Itgal/Smad3/B2m/Spi1/Ranbp1/Ccna2/Mapk10/Akt1/Tgfb3/Ccnb2/Nrp1/Tgfb1/Ets1/Chek2/H2-M3/Adcy7/Il2rg/Ccnd1/Itgb2/H2-DMa/Ccnd2/Pttg1/Il15/H2-Eb1/H2-Ab1/H2-Aa | 219 | 0.0268191168272031 |
| mmu04141 | Protein processing in endoplasmic reticulum - Mus musculus (house mouse) | Hspa1l/Herpud1/Dnajc1/Hspa1a/Ppp1r15a/Xbp1/Dnajc3/Rbx1/Hsp90aa1/Atf6/Ube2g2/Eif2s1/Ngly1/Ubxn4/Map3k5/Sec63/Dnajb12/Syvn1/Ubqln2/Sil1/Hspa1b/Sec24b/Cul1/Dnajb2/Bcl2/Nploc4/Fbxo6/Dnajb11/Ubxn8/Hsp90b1/Sec62/Fbxo2/Preb/Ubqln4/Sel1l2/Ube2j1/Nfe2l2/Yod1/Os9/Atf4/Rpn1/Ubxn6/Sar1a/Hyou1/Hspa8/Mbtps2/Hsp90ab1/Capn2/Ubxn1/Nsfl1c/Wfs1/Ubqln1/Hspa4l/Bax/Lman1l/Uggt1/Hspa5/Derl2/Ube4b | 157 | 0.0274382114403415 |
| mmu04216 | Ferroptosis - Mus musculus (house mouse) | Hmox1/Slc39a8/Cp/Gclm/Slc3a2/Map1lc3b/Slc40a1/Lpcat3/Sat1/Prnp/Slc39a14/Acsl3/Gss/Fth1/Gclc | 37 | 0.0310336714002967 |
| mmu04115 | p53 signaling pathway - Mus musculus (house mouse) | Cdkn1a/Serpine1/Bcl2l1/Fas/Sesn1/Igfbp3/Ddb2/Ccng2/Mdm4/Gadd45g/Gadd45b/Siah1a/Ccnb1/Bcl2/Pten/Perp/Atr | 71 | 0.0312073474054257 |
| mmu04136 | Autophagy - other - Mus musculus (house mouse) | Gabarapl1/Atg13/Atg101/Pik3c3/Mtor/Wipi2/Ppp2ca/Igbp1/Atg9a/Gabarapl2/Atg2a/Ppp2cb/Atg2b | 32 | 0.0326704684150803 |
| mmu04070 | Phosphatidylinositol signaling system - Mus musculus (house mouse) | Ppip5k2/Ppip5k1/Pik3r1/Itpkc/Dgka/Plcb4/Itpr2/Mtmr14/Mtmr4/Pik3c3/Itpk1/Inpp4b/Inpp4a/Plcg1/Mtm1/Pi4k2a/Ocrl/Ip6k2/Mtmr1/Pten/Pi4kb/Cds2/Pik3ca/Plcb2/Inpp5f/Synj1/Ip6k1/Prkcg/Dgkh/Mtmr7/Mtmr2/Pip4k2c/Pikfyve/Dgki/Pik3cb/Pik3c2g | 88 | 0.0319954587370827 |
| mmu04360 | Axon guidance - Mus musculus (house mouse) | Pak6/Unc5b/Sema3f/Camk2a/Src/Rock2/Smo/Fes/Ntn3/L1cam/Plxna3/Epha6/Hras/Ppp3cb/Nck2/Cdc42/Epha7/Slit1/Kras/Pard6a/Ptk2/Lrrc4c/Ilk/Sema6a/Sema4a/Sema6d/Plxnb1/Mapk3/Camk2g/Plxnb3/Fyn/Pik3r2/Ephb1/Pik3r3/Myl12b/Prkca/Sema3b/Myl9/Gnai2/Wnt5b/Plxna1/Epha4/Plxnc1/Met/Plcg2/Abl1/Efna1/Rgma/Dpysl2/Trpc3/Wnt5a/Cxcl12/Nrp1/Rac2/Cfl1/Ntn4/Ephb3/Slit2/Cxcr4/Sema3c/Efnb3 | 178 | 0.0324222000202192 |
| mmu05017 | Spinocerebellar ataxia - Mus musculus (house mouse) | Reln/Pik3r1/Atg13/Psmd4/Psmd7/Xbp1/Atg101/Vldlr/Plcb4/Itpr2/Psmc1/Pik3c3/Psmd11/Mtor/Psmc5/Rora/Map3k5/Kat5/Psmd2/Rb1cc1/Wipi2/Sp1/Psmd1/Tbp/Psmd6/Psmd12/Psmc4/Psma7/Ambra1/Psmc3/Pik3ca/Plcb2/Psmd13/Grin3b/Psma6/Psma8/Prkcg/Fgf14/Gtf2b/Adrm1/Atg2a/Cyct/Atxn10/Cic/Atg2b/Psmc2/Pik3cb/Grin2c/Pum1/Gria3/Ern1/Dab1/Psma3/Atxn2l | 138 | 0.0335130061839963 |
| mmu03010 | Ribosome - Mus musculus (house mouse) | Mrpl32/Mrpl21/Rpsa/Mrpl1/Mrpl20/Rpl3l/Mrpl22/Mrpl10/Mrps2/Mrps6/Mrpl18/Mrps17/Mrpl13/Mrps14/Mrpl35/Mrpl14/Mrps16/Mrps10/Mrpl27/Mrps11/Mrpl16/Mrpl4/Mrpl28/Rpl39l/Mrpl12/Mrpl34/Mrpl19 | 96 | 0.0355261895924463 |
| mmu00270 | Cysteine and methionine metabolism - Mus musculus (house mouse) | Cdo1/Gclm/Ahcy/Psat1/Gnmt | 48 | 0.038207989859622 |
| mmu04664 | Fc epsilon RI signaling pathway - Mus musculus (house mouse) | Hras/Map2k2/Sos2/Lcp2/Kras/Mapk11/Mapk3/Fyn/Pik3r2/Pik3r3/Mapk9/Grb2/Prkca/Alox5ap/Plcg2/Vav3/Mapk10/Btk/Akt1/Rac2/Alox5/Fcer1g/Pla2g4a/Map2k6 | 64 | 0.0382435271814953 |
| mmu05143 | African trypanosomiasis - Mus musculus (house mouse) | Sele/Ido1/F2rl1/Prkca/Il18/Il1b/Ido2/Lama4/Prkcb/Vcam1/Hbb-b2 | 33 | 0.0421559670931643 |
| mmu00280 | Valine, leucine and isoleucine degradation - Mus musculus (house mouse) | Hmgcs2/Aox1/Aldh2/Ehhadh/Abat/Ivd/Acadsb/Aldh3a2 | 51 | 0.0443596189037632 |
| mmu04978 | Mineral absorption - Mus musculus (house mouse) | Mt2/Hmox1/Mt1/Slc40a1/Atp7a/Slc5a1 | 49 | 0.0457707576222176 |
| mmu05169 | Epstein-Barr virus infection - Mus musculus (house mouse) | Ifnar2/E2f1/Nfkb1/Irak1/H2-T3/Nfkbie/Psmd14/H2-Q10/Mapk11/Casp3/Cd19/Cr2/H2-Oa/Cxcl10/Oas3/Pik3r2/Pik3r3/Mapk9/H2-D1/Casp8/Tnfaip3/Sap30/Cd3g/Ifna11/H2-K1/Cdk4/E2f2/Tlr2/Trp53/Relb/Tap1/Itgal/B2m/Bak1/Eif2ak2/Entpd8/Plcg2/Ccna2/Mapk10/Btk/Akt1/Irf9/H2-M3/Irf7/Traf3/Oas1a/Gadd45a/Ccnd1/Vim/Blnk/Map2k6/H2-DMa/Ccnd2/Stat1/H2-Eb1/H2-Ab1/H2-Aa | 190 | 0.0464136862379526 |
| mmu05219 | Bladder cancer - Mus musculus (house mouse) | Cdkn1a/Hbegf/Fgfr3/Myc/Mmp9/Braf/Rb1/Erbb2/Dapk2/Araf/Dapk3/Dapk1/Rassf1 | 40 | 0.0506145264156384 |
| mmu05146 | Amoebiasis - Mus musculus (house mouse) | Actn4/Gna14/Nfkb1/Col4a4/C8g/Lamb1/Ptk2/Prkaca/Casp3/Serpinb6e/Pik3r2/Pik3r3/Lama3/Itgam/Prkca/Il1b/Lama2/Adcy1/Tlr2/Rab5c/Tgfb3/Tgfb1/Lama4/Prkcb/Fn1/Col1a1/Col1a2/Itgb2/Lamb3/Col3a1 | 98 | 0.0499040307101727 |
